# Identification of new components of the basal pole of *Toxoplasma gondii* provides novel insights into its molecular organization and functions

**DOI:** 10.3389/fcimb.2022.1010038

**Published:** 2022-10-13

**Authors:** Chloé Roumégous, Aya Abou Hammoud, Damien Fuster, Jean-William Dupuy, Corinne Blancard, Bénédicte Salin, Derrick R. Robinson, Patricia Renesto, Isabelle Tardieux, Karine Frénal

**Affiliations:** ^1^ Univ. Bordeaux, CNRS, Microbiologie Fondamentale et Pathogénicité, UMR 5234, Bordeaux, France; ^2^ Univ. Bordeaux, Plateforme Protéome, Bordeaux, France; ^3^ Univ. Bordeaux, CNRS, Institut de Biochimie et Génétique Cellulaires, UMR 5095, Bordeaux, France; ^4^ IAB, Team Biomechanics of Host-Apicomplexa Parasite, INSERM U1209, CNRS UMR5309, Grenoble Alpes University, Grenoble, France

**Keywords:** Apicomplexa, *Toxoplasma gondii*, cytoskeleton, basal complex, myosin heavy chain (MHC), constriction, cell-cell communication, expansion microscopy

## Abstract

The *Toxoplasma gondii* tachyzoite is a singled-cell obligate intracellular parasite responsible for the acute phase of toxoplasmosis. This polarized cell exhibits an apical complex, a hallmark of the phylum Apicomplexa, essential for motility, invasion, and egress from the host cell. Located on the opposite end of the cell is the basal complex, an elaborated cytoskeletal structure that also plays critical roles in the lytic cycle of the parasite, being involved in motility, cell division, constriction and cytokinesis, as well as intravacuolar cell-cell communication. Nevertheless, only a few proteins of this structure have been described and functionally assessed. In this study, we used spatial proteomics to identify new basal complex components (BCC), and *in situ* imaging, including ultrastructure expansion microscopy, to position them. We thus confirmed the localization of nine BCCs out of the 12 selected candidates and assigned them to different sub-compartments of the basal complex, including two new domains located above the basal ring and below the posterior cup. Their functional investigation revealed that none of these BCCs are essential for parasite growth *in vitro*. However, one BCC is critical for constricting of the basal complex, likely through direct interaction with the class VI myosin heavy chain J (MyoJ), and for gliding motility. Four other BCCs, including a phosphatase and a guanylate-binding protein, are involved in the formation and/or maintenance of the intravacuolar parasite connection, which is required for the rosette organization and synchronicity of cell division.

## Introduction

*Toxoplasma gondii* is a unicellular human and animal pathogen belonging to the Apicomplexa. This phylum also includes other obligate intracellular parasites of medical importance, such as *Plasmodium*, the causative agent of malaria responsible for more than half-million deaths per year (Global Malaria Programme, 2021) or *Cryptosporidium*, a leading cause of severe diarrhea in young children (Kotloff et al., 2013). *T. gondii* is responsible for toxoplasmosis. This foodborne disease is primarily harmless but can lead to severe outcomes in immunocompromised individuals or for fetuses in case of primary infection during pregnancy. Most serious pathologies are related to the capacity of *T. gondii* to establish and persist in tissues. In humans, it is estimated that about one-third of the population carries the parasite, with significant disparity worldwide (Hakimi et al., 2017).

The tachyzoite is the fast-replicative stage of *T. gondii*, responsible for the acute phase of the disease. It is motile and can invade and multiply in any nucleated cell leading to their lysis upon egress (Blader et al., 2015). This highly polarized cell displays an apicomplexan-specific apical complex composed of a unique cytoskeletal structure, the conoid, and two sets of secretory organelles, the micronemes, and rhoptries, which sequentially discharge their content during the invasion of the host cell. Because of its essential role in parasite motility and invasion, the apical complex has been the subject of intensive research for decades [recently reviewed in (Dubois and Soldati-Favre, 2019; Dos Santos Pacheco et al., 2020; Lebrun et al., 2020; Ben Chaabene et al., 2021)]. In contrast, and located at the opposite pole of the cell, the basal complex is significantly less documented, while an increasing volume of evidence argues for its critical roles all along the lytic cycle of the parasite (Morano and Dvorin, 2021; Gubbels et al., 2022).

The basal complex is a highly organized structure composed of juxtaposed rings located below the basal end of the inner membrane complex (IMC) that runs underneath the plasma membrane for most of the parasite length (Hu, 2008). The IMC is a membranous structure made of flattened vesicles, joined by transverse and longitudinal sutures, and a network of alveolin domain-containing proteins resembling the metazoan’s intermediate filaments (Mann and Beckers, 2001; Gould et al., 2008; Anderson-White et al., 2011; Chen et al., 2017). Based on immunofluorescence studies, the basal complex is composed of a basal ring located at the edge of the IMC and a structure below, known as the posterior cup. According to electron micrographs, the basal ring appears even more complex, composed of two interwoven rings named inner ring and inner collar (Anderson-White et al., 2011).

In extracellular parasites, the basal complex is involved in gliding motility. Alternating with the apical pole, it constitutes a periodic adhesion/de-adhesion site for force transmission during parasite movement (Pavlou et al., 2020). A glideosome complex comprising the class XIV myosin heavy chain C (MyoC) has been described at the basal ring with the capacity to redistribute underneath the plasma membrane to assist actin-based motility (Frénal et al., 2014). In intracellular parasites, throughout the division, the basal complex is part of the scaffold necessary for developing new polarized progenies that grow by internal budding within the mother cell through an endodyogeny process (Gubbels et al., 2006; Hu, 2008). Remarkably, the basal complex of the developing daughter cells assembles very early during division, with one of its components, MORN1, appearing at the time of centrosome duplication, forming a ring-shaped structure that caps the edge of the progeny IMC along its extension and eventually constricts and segregates the mature parasites (Gubbels et al., 2006; Hu, 2008; Heaslip et al., 2010; Lorestani et al., 2010). MORN1 likely acts as a scaffolding protein during cell division, being able to self-assemble as a ring (Heaslip et al., 2010). Its disruption leads to a defect in basal complex assembly, constriction, and successful segmentation of the progenies (Heaslip et al., 2010; Lorestani et al., 2010). The same phenotype was observed following the depletion of the recently described basal complex component 4 [BCC4, (Engelberg et al., 2022)] or the haloacid dehalogenase (HAD) phosphatase HAD2a, although the substrates of this enzyme remain unknown (Engelberg et al., 2016). Surprisingly, actin (ACT1) is not required for cell division but is critical for constricting the newly formed mature parasites (Periz et al., 2017). Indeed, a larger basal complex is observed upon disruption of ACT1 but also upon depletion of two proteins located at the posterior cup, the class VI myosin heavy chain J (MyoJ) or the CaM-like protein centrin 2 (CEN2), giving the parasite a “torpedo” shape (Frénal et al., 2017; Periz et al., 2017; Lentini et al., 2019). These results suggest that an actomyosin system resembling a contractile ring machinery and located at the basal cup exists in Apicomplexa. However, none of these proteins is homologous to the contractile ring involved in the division of higher eukaryotes such as *Saccharomyces cerevisiae* or *Drosophila melanogaster* and *vice versa* (Morano and Dvorin, 2021). Finally, at the end of each round of division, the basal poles of the mature parasites remain connected to a residual body *via* cytoplasmic bridges that allow the diffusion of soluble proteins and metabolites between cells and so ensure their fast proliferation by synchronous rounds of division (Frénal et al., 2017). The formation and/or maintenance of these connections require the presence of the myosin heavy chain MyoI (Frénal et al., 2017). An extensive filamentous actin network also fills these cell-cell connections and is dismantled along with the multistep process that leads to the active egress of the tachyzoite from the host cell (Periz et al., 2017; Li et al., 2022). It remains unknown how the tachyzoites orchestrate the timely disruption of these connections to fully contract their basal complexes and seal their plasma membranes to allow efficient egress and motility.

To get a deeper understanding of the molecular composition, organization, and function(s) of the basal complex, we carried out biotin-based proximity labeling during the last hours of the tachyzoite lytic cycle to identify new basal complex proteins using the basal cup protein MyoJ as bait. We identified more than 100 highly enriched proteins and selected 12 for further analyses. We confirmed that nine of them are located in different basal complex sub-compartments and also performed their functional characterization during the lytic cycle of the tachyzoite. Using optical and super-resolution microscopy, we identified two additional sub-compartments of the basal complex, one lying above the basal ring nearby the IMC and one under the posterior cup at the border with the intravacuolar nanotubes connecting the parasites. We determined that BCC1 is critical for constricting the basal pole of the tachyzoite and for their gliding motility. We additionally show that depletion of either BCC1 or MyoJ causes the destabilization of the other one, suggesting that BCC1 directly interacts with MyoJ, likely acting as cargo to carry the motor to the basal cup. Furthermore, we identified four BCCs (BCC5, BCC8, BCC10, and BCC11) playing a role in the formation and/or the maintenance of the intravacuolar connection. Down-regulation of these proteins impacts the rosette organization of the parasites and their synchronicity of division.

## Materials and methods

### Cloning of DNA constructs

All genomic DNA (gDNA) have been prepared from tachyzoites using either DNAzol reagent (Invitrogen) or the NucleoSpin DNA RapidLyse kit (Macherey-Nagel) according to manufacturer instructions. All primers were synthesized by Integrated DNA Technologies (IDT) and are listed in **Supplementary Table 1**. All amplifications for cloning were performed with the Q5 High-Fidelity DNA Polymerase (New England Biolabs, NEB) while PCRs for screening were performed with the Taq DNA polymerase with the ThermoPol Buffer (NEB). All restriction enzymes used were from NEB.

For the endogenous tagging of MyoJ with the TurboID enzymatic domain, the knock-in vector pKI-MyoJ-CT-TurboID-3Ty_HXGPRT has been generated. The sequence corresponding to the TurboID was amplified by PCR using the plasmid HA-TurboID-NLS-pCDNA3 (Addgene #107171) as template and the primers Turbo-8271/Turbo-8270. This insert was digested with NsiI/SbfI restriction enzymes and cloned into the pKI-MyoJ-3Ty_HXGPRT vector (Frénal et al., 2017) digested with NsiI and dephosphorylated. The vector has been linearized with XcmI prior transfection of the parasites.

For the endogenous tagging of Candidate 3, the pKI-Candidate3-4Myc-DHFR vector has been cloned. A region of gDNA corresponding to the 3’ end of the *TGGT1_273905* gene was amplified using the primer pair MIP3-1760/MIP3-1761 and cloned by AQUA cloning (Beyer et al., 2015) into the vector pTub8-MIC13-4Myc_DHFR (gift from Dominique Soldati-Favre) digested with ApaI and NsiI. The vector was linearized using BstEII for stable transfection.

A specific sgRNA vector targeting the 3’ end of *MyoJ* has been generated using the Q5 site-directed mutagenesis kit (New England Biolabs) with the vector pSAG1::CAS9-GFP-U6::sgUPRT as template (gift from David L. Sibley, [Shen et al., 2014)] and the primer pair TgMyoJ-1734/gRNA-4883. For each candidate, a specific sgRNA vector targeting the 3’ end of the corresponding gene has been generated using the pU6-Universal vector [gift from Sebastian Lourido, (Sidik et al., 2014)] as backbone. For each sgRNA, two primers have been annealed together and ligated into the pU6-Universal vector digested by BsaI restriction enzyme.

### *Toxoplasma gondii* tachyzoite cultures

*T. gondii* tachyzoite strains were grown in confluent human foreskin fibroblasts (HFF, ATCC CRL-1634) maintained in Dulbecco´s Modified Eagle´s Medium (Gibco DMEM) supplemented with 5% fetal calf serum, 2 mM glutamine and 25 μg/ml gentamicin.

Parasite transfections were performed with either the Nucleofector 2b Device (Amaxa) or the Gene Pulser Xcell Total System (Bio-Rad). For transfection with the Amaxa device, about 10^6^ parasites from RHΔKU80 (Huynh and Carruthers, 2009) and derivative strains, including the TIR1-3FLAG strain (Long et al., 2017), were resuspended in 100 μl of parasite nucleofector solution from the Basic Parasite Nucleofector Kit 2 (Lonza) and electroporated using the program U-033. For transfection with the Bio-Rad device, the same quantity of parasites was resuspended in 500 μl of cytomix supplemented with ATP (2 mM) and GSH (5 mM) and electroporated using the exponential wave with the following settings: voltage 1700 V, resistor 50 Ω, capacitor 25 µF (Jacot et al., 2014).

For the knock-in insertion with linearized vector, 15 μg of linearized plasmid was used. For CRISPR-Cas9 mediated recombination, 15 μg of the circular sgRNA-specific vector was transfected together with 10-15 μg of PCR products containing approximatively 32 bp homology with the targeted locus. All PCR products were produced with the Q5 High-Fidelity DNA Polymerase (NEB) and the primers listed in **Supplementary Table 1**.

For testing a potential co-localization with filamentous actin, parasites were engineered to transiently express anti-F-actin chromobodies (Periz et al., 2017) post transfection with 30 μg of circular pTub8-ACT-Cb-GFP vector (gift from Dominique Soldati-Favre) (Tosetti et al., 2019).

Mycophenolic acid (MPA, 25 μg/mL) and xanthine (50 μg/mL) or pyrimethamine (1 µg/ml) were used to select the resistant parasites carrying the HXGPRT and DHFR cassette, respectively. 500 μM of auxin (or IAA for Indole-3-acetic acid, Sigma-Aldrich) was used for conditional down-regulation of mAID cell lines.

### Streptavidin pull-down and mass spectrometry

Confluent HFFs were highly infected with MyoJ-TurboID-3Ty or the parental RHΔKU80 strain and the parasites were allowed to grow for about 40 h. After a wash with complete medium to remove all the extracellular parasites, the biotinylation was performed for 7 h by addition of 150 μM of biotin (Sigma-Aldrich) into the medium. At the time of collecting the parasites, the HFF monolayer was almost totally disrupted, and the parasites were almost all extracellular. The samples were washed three times with large volumes of cold PBS and frozen at -80°C. The MyoJ-TurboID-3Ty samples were performed in experimental duplicates. The pellets were resuspended in RIPA buffer (50 mM Tris pH 7.5, 150 mM NaCl, 0.1% SDS, 1% Triton X-100, 0.5% Sodium Deoxycholate) supplemented with the protease inhibitor cocktail Set I (Calbiochem), incubated for 30 min on ice and then sonicated (Diagenode bioruptor, 5 cycles: 15 s ON followed by 30 s OFF). The total protein content of the samples was quantified at 2.1 ± 0.3 mg. The lysates were then centrifuged at 14,000 x g for 30 min at 4°C to pellet insoluble material. In parallel, 200 μl of unsettled magnetic streptavidin beads (Bio-Adembeads, 300 nm) were equilibrated in RIPA buffer. Supernatants were then added onto the streptavidin beads and incubated for 2 h at room temperature with gentle agitation. Beads were washed three times in RIPA buffer, followed by one wash in 8 M Urea buffer (Urea 8 M, 50 mM Tris-HCl pH 7.4, 150 mM NaCl), one wash in RIPA buffer and finally two washes in PBS. Beads were boiled in Laemmli buffer and centrifuged. 10% of the supernatant was collected for western-blot analysis and the remaining was analyzed by mass spectrometry.

For mass spectrometry analyses, the supernatants were loaded on a 10% acrylamide SDS-PAGE gel, and migration was stopped when samples had just entered the resolving gel. Proteins were visualized by colloidal blue staining and each unresolved region of the gel was removed as one segment. Sample preparation and trypsin digestion of the proteins were performed as previously described (Allmann et al., 2014). NanoLC-MS/MS analyses were performed using an Ultimate 3000 RSLC Nano-UPHLC system (Thermo Scientific, USA) coupled to a nanospray Orbitrap Fusion™ Lumos™ Tribrid™ Mass Spectrometer (Thermo Scientific, USA). The peptide extracts were loaded on a 300 µm ID x 5 mm PepMap C18 precolumn (Thermo Scientific, USA) at a flow rate of 10 µL/min. After a 3 min desalting step, peptides were separated on a 50 cm EasySpray column (75 µm ID, 2 µm C18 beads, 100 Å pore size, ES803A rev.2, Thermo Fisher Scientific) with a 4-40% linear gradient of solvent B (0.1% formic acid in 80% ACN) in 48 min. The separation flow rate was set at 300 nL/min. The mass spectrometer operated in positive ion mode at a 2.0 kV needle voltage. Data were acquired using Xcalibur 4.1 software in a data-dependent mode. MS scans (m/z 375-1500) were recorded at a resolution of R = 120000 (@ m/z 200) and an AGC target of 4×10^5^ ions was collected within 50 ms, followed by a top speed duty cycle of up to 3 s for MS/MS acquisition. Precursor ions (2 to 7 charge states) were isolated in the quadrupole with a mass window of 1.6 Th and fragmented with HCD@30% normalized collision energy. MS/MS data were acquired in the ion trap with rapid scan mode, AGC target of 3x10^3^ ions, and a maximum injection time of 35 ms. Selected precursors were excluded for 60 s. Protein identification and Label-Free Quantification (LFQ) were done in Proteome Discoverer 2.4. MS Amanda 2.0, Sequest HT, and Mascot 2.5 algorithms were used for protein identification in batch mode by searching against a *Toxoplasma gondii* GT1 strain protein database (8 459 entries, release 48, https://toxodb.org/website). Two missed enzyme cleavages were allowed for the trypsin. Mass tolerances in MS and MS/MS were set to 10 ppm and 0.6 Da. Oxidation (M), acetylation (K), and biotinylation (K) were searched as dynamic modifications, and carbamidomethylation (C) as a static modification. Peptide validation was performed using Percolator algorithm (Käll et al., 2007) and only “high confidence” peptides were retained corresponding to a 1% false discovery rate at the peptide level. Minora feature detector node (LFQ) was used along with the feature mapper and precursor ions quantifier. The quantification parameters were selected as follows: (1) Unique peptides (2) Precursor abundance based on intensity (3) No normalization was applied (4) Protein abundance calculation: summed abundances (5) Protein ratio calculation: pairwise ratio based and (6) Hypothesis test: t-test (background based). Quantitative data were considered for master proteins, quantified by a minimum of 2 unique peptides, fold changes above 2, and a statistical p-value lower than 0.05. The mass spectrometry proteomics data have been deposited to the ProteomeXchange Consortium *via* the PRIDE (Deutsch et al., 2020) partner repository with the dataset identifier PXD035106.

### Immunofluorescence assay (IFA)

Infected HFF cells seeded on coverslips were washed in PBS prior to 10 min fixation with 4% paraformaldehyde (PFA) in PBS. Cells were then rinsed in PBS and 0.1 M glycine and permeabilized using Triton X-100 at 0.2% in PBS for 30 min. Non-specific binding sites were blocked in the same buffer supplemented with 2% BSA for another 30 min. Primary antibodies diluted in PBS and 2% BSA were added to slides for 1 h. Cells were then washed in PBS and Triton X-100 0.2% before 1 h incubation with secondary antibodies diluted in PBS and 2% BSA. For triple labeling, cells were first labeled with α-Ty (mouse) for 1 h followed by its corresponding secondary antibody for 1 h, and then probed with α-HA (rat)/α-IMC1 (rabbit) followed by their corresponding secondary antibodies. All the primary and secondary antibodies used are listed in **Supplementary Table 2**.

Wide-field images were acquired on a Zeiss Axioplan microscope with Zeiss 63x or 100x objectives (NA 1.4), using a Photometrics Coolsnap HQ2 camera and Metamorph software (Molecular Devices, San Jose, CA, USA). Confocal images were collected at the Bordeaux Imaging Center with a Leica TCS SP5 on an upright stand DM6000 (Leica Microsystems, Mannheim, Germany), using objectives HCX Plan Apo CS 63X oil NA 1.40. For confocal microscopy the lasers used were Diode 405 nm, Argon (458 nm, 476 nm, 488 nm, 496 nm, 514 nm) Green Helium-Neon 561 nm and Red Helium-Neon 633 nm. The system was equipped with a conventional scanner (10Hz to 2800 Hz) and 5 internal detectors, (3 conventional PMT and 2 hybrid detectors), 2 non-descanned hybrid detectors, and 1 PMT for transmission. All images were processed and analyzed using ImageJ and Fiji software (Schindelin et al., 2012; Schneider et al., 2012).

### Plaque assays

Plaque assays have been performed on 6-well plates by inoculating freshly egressed parasites on confluent HFF cells. The parasites were then allowed to grow for 7 days with or without auxin (IAA). At day 7, the cells have been fixed for 10 min with PBS containing 4% PFA and 0.05% glutaraldehyde (GA) and then stained with crystal violet as previously described (Coleman and Gubbels, 2012).

### Competition assays

BCC1-mAID-3HA/MyoJ-2Ty parasites were mixed with GFP-expressing parasites at a ratio of about 1:1. This ratio was then determined over 5 passages (14 days) by IFA using α-GAP45. At each passage, 100 vacuoles were counted in duplicate from three independent biological replicates. The ratios have been normalized to 50% at t0. The data are presented as mean ± SD.

### Gliding assay (trail deposition assay)

Freshly egressed parasites from the BCC1-mAID-3HA/MyoJ-2Ty, MyoJ-mAID-3HA/BCC1-2Ty and TIR1 strains pre-treated for 24 h with or without IAA prior to egress were centrifuged at 1000 g for 10 min and resuspended in a calcium saline solution. A drop of sample was then placed onto Poly-L-Lysine coated coverslips and the parasites were allowed to glide at 37°C for 10 min before fixation with 4% paraformaldehyde/0.05% glutaraldehyde in PBS for 10-15 min. Trails were detected by immunofluorescence assays using a hybridoma of α-SAG1 antibodies. 1ml of calcium saline solution is composed of: 0.5 ml of 2x HEPES buffer, 50 μl of 100mM CaCl_2_ (5mM) and 450 μl of sterile water. The 2x HEPES buffer is made of: NaCl (274 mM), KCl (10 mM), Na_2_HPO_4_ (2 mM), glucose (11 mM) and HEPES (42 mM), pH 7.05. The primary and secondary antibodies used are listed in **Supplementary Table 2**.

The percentage of motility was calculated by counting the total number of trails (circular, helical and short) and the total number of parasites per field for 6 to 10 fields per condition, in duplicates, and for four independent experiments. The percentage of each type of motility (circular or helical) was determined for the same fields of the four independent experiments. An average of 195±87 parasites and 74±45 trails per sample were counted. The length of the helical trails was measured using the segmented line tool of Image J by counting 17 ± 3 trails per condition, for three independent experiments. The results are presented as mean ± SD, except the length of the trails presented as mean ± SEM. Their significance was assessed using an unpaired t-test (t-test calculator from GraphPad), and the two-tailed p-values are written on the graphs.

### Videomicroscopy

Human osteosarcoma cells (U2OS) cells obtained from Sigma-Aldrich were plated on 18 mm diameter and poly-L-lysine -coated glass coverslips. The next day, cells were infected with a single parasite and incubated for 36 to 42 h at 37°C under a 5% CO2 atmosphere to allow several cycles of parasite multiplication. Samples were then installed on an appropriate chamber that fits the platform of the Eclipse Ti inverted confocal microscope (Nikon France Instruments, Champigny sur Marne, France) coupled to a CMOS Prime camera (Photometrics, Tucson, AZ, USA) and a CSU X1 spinning disk (Yokogawa Roper Scientific, Lisses, France). To select for cells ready to spontaneously release the tachyzoite progeny, images were captured with a 60x Plan-neofluar objective (NA: 1.46) using the multi-stage position in the MetaMorph software (Molecular Devices, Sunnyvale, USA). When time was appropriate, a single position was recorded at about 1 frame/s for 20 min to follow parasite behavior at the time of egress and immediately after. Data were processed using both MetaMorph software and Fiji (Schindelin et al., 2012) as previously described (Vigetti et al., 2022).

### Fluorescence recovery after photobleaching (FRAP)

30 μg of circular pTub8-GFP-HXGPRT (Hettmann et al., 2000) vector were used for transient transfection of the cell lines of interest. Transfected parasites were then allowed to grow on confluent HFF cells for 24-30 h with or without auxin (500 µM). FRAP experiment were performed at the Bordeaux Imaging Center on a spinning disk microscope Leica DMI8 (Leica Microsystems, Wetzlar, Germany) equipped with a confocal Scanner Unit CSU-W1 T2 (Yokogawa Electric Corporation, Tokyo, Japan) using an HCX PL Apo CS2 63X oil NA 1.4. The 37°C atmosphere was created with an incubator box and an air heating system (PeCon GmbH, Germany). This system was controlled by MetaMorph software (Molecular Devices, Sunnyvale, USA). Images were acquired using a sCMOS Prime 95B camera (Photometrics, Tucson, USA). The LASER diode used was at 488 nm (400 mW). Photo-manipulation experiments were done with an iLas² scanner system (Gataca Systems, Massy, France) using the same Laser bench as for imaging. At least one parasite per GFP-expressing vacuole was photobleached with 10 pulses at 100% of the 488 nm laser power. Recovery from photobleaching was monitored by 20 consecutive acquisitions at 1 image per second followed by 1 image every 10 or 20 s for 180 s. Images were analyzed using home-made ImageJ script developed by Fabrice Cordelières from the Bordeaux Imaging Center (https://github.com/fabricecordelieres/IJ-Macro_FRAP-MM).

### Ultrastructure expansion microscopy (U-ExM)

U-ExM experiments have been carried out following the protocol described in Dos Santos Pacheco and Soldati-Favre, 2021 (Dos Santos Pacheco and Soldati-Favre, 2021) with the following modifications. Incubation in the formaldehyde/acrylamide mix for protein anchoring and crosslinking prevention was performed either for 3 h or overnight at 37°C. For the first expansion, gels were bathed three times in 30 to 100 mL of water. Gels were then cut in 8 mm diameter circles and stained with primary antibodies for 3 h or overnight at room temperature. During secondary antibodies incubation, DNA was stained using Hoechst for 1 h at room temperature. Gel mounting for imaging has been performed as described previously (Casas et al., 2022). For intracellular parasites preparation, HFF cells were settled on coverslips in order to reach 50 to 70% confluence prior the infection. After 24 h, cells were washed in PBS and the previous protocol, described above, was followed. The expansion factor was assessed by measuring the gels pre- and post-expansion. Only the gels with an expansion factor > 3,9 were further processed for labeling and imaging. Images were acquired using the confocal microscope Leica TCS SP5 as described above. All the primary and secondary antibodies used are listed in **Supplementary Table 2**.

### Electron microscopy

Parasite-infected HFF cells were fixed for 45 min at 4°C in 4% glutaraldehyde in PBS, washed and fixed again 1 h at room temperature in 2% osmium tetroxide in PBS containing 15 mg/ml of K_4_Fe(CN)_6_. Dehydration was performed with ethanol series (50%, 70%, 95% and absolute ethanol). Next, the samples were embedded progressively in Epon resin. Ultrathin sections were contrasted with 1% lead citrate in water for 1 min. Sample imaging was performed using a Hitachi H7650 transmission microscope operated at 80 KV with a camera Gatan - 11 MPx at the electron microscopy unit of the Bordeaux Imaging Center.

### Western blot

Parasites were lysed in RIPA buffer (50 mM Tris pH 7.5, 150 mM NaCl, 0.1% SDS, 1% Triton X-100, 0.5% Sodium Deoxycholate) and sonicated at 4°C (Diagenode bioruptor, 5 cycles: 15 s ON followed by 30 s OFF). Lysates were then incubated on ice 20 min and then centrifuged at 4°C for 30 min at 21 000 g. Supernatants were mixed with 4x Laemmli sample buffer and loaded on 8% acrylamide/bisacrylamide SDS-PAGE gels. Proteins were transferred on a nitrocellulose membrane using a wet transfer system (Bio-Rad). Non-specific binding sites were blocked by 5% non-fat milk in TBS/0.2% Tween20. Membranes were probed with antibodies diluted in the same buffer. HRP-conjugated antibodies were visualized using ImageQuant (LAS 4000, General Electric) and fluorophore-conjugated antibodies, using ChemiDoc (Bio-Rad). All the primary and secondary antibodies used are listed in **Supplementary Table 2**.

## Results

### Identification of new basal complex candidates by proximity labeling

**Figure 1:**
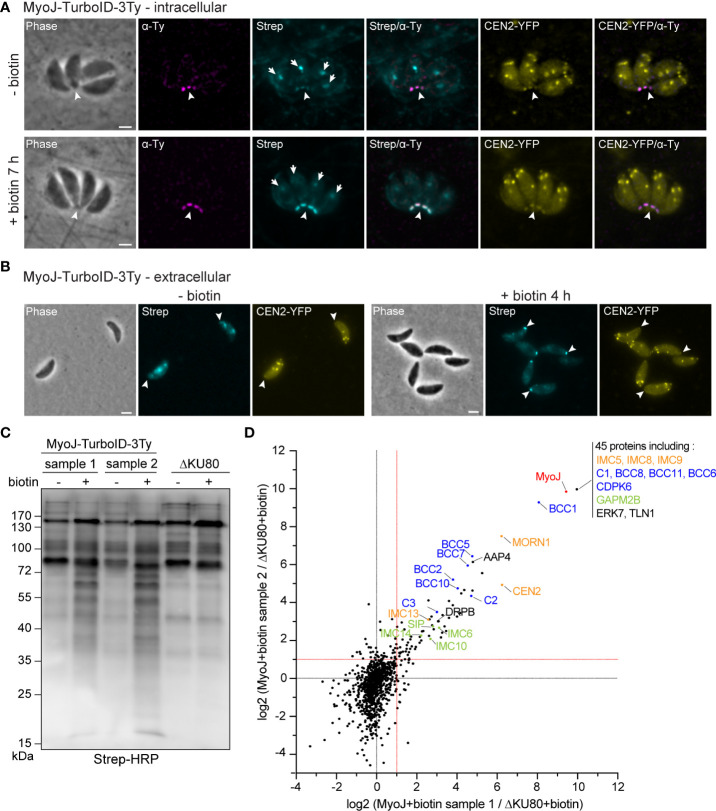
Identification of putative MyoJ-interacting proteins using TurboID. Immuno-detection of MyoJ endogenously tagged with TurboID-3Ty (MyoJ-TurboID-3Ty) in intracellular **(A)** and extracellular **(B)** tachyzoites also expressing endogenously tagged CEN2 (CEN2-YFP). MyoJ-TurboID-3Ty localizes at the basal end of the parasite (arrowhead). Biotinylated proteins are detected using a fluorophore-conjugated streptavidin (Strep). In absence of biotin, mainly the apicoplast (arrow) of each parasite is labelled with a weak signal at the posterior pole. In contrast, after incubation with biotin, mainly the posterior pole is stained. Magenta: mouse anti-Ty, Cyan: streptavidin-AlexaFluor 594, Yellow: CEN2-YFP. Scale bars: 2 μm. **(C)** Western blot of biotinylated proteins, isolated with streptavidin magnetic beads after incubation of egressing ΔKU80 and MyoJ-TurboID-3Ty parasites ± biotin for 7 h. Revelation was performed using streptavidin-HRP. **(D)** Scatter plot of the log2(ratio) of the proteins found in the two replicates (samples 1 and 2). The bait MyoJ is shown in red, the candidates investigated in this study (CX and BCCX) are in blue, the known basal complex proteins in orange, the known IMC proteins in green, and the other known proteins in black.

To expand our knowledge about the composition of the basal complex, proximity labeling experiments were performed using the TurboID, a promiscuous mutant of *Escherichia coli* biotin ligase BirA, that speeds up biotinylation of the neighboring proteins (Branon et al., 2018). To do so, MyoJ was endogenously fused to the TurboID and 3Ty-tags by a knock-in strategy into the endogenously tagged CEN2-YFP cell line (**Supplementary Figure 1**). The fusion protein was correctly targeted to the basal cup of the parasite, as shown by its superposed localization with the basal signal of CEN2 (**Figure 1A**). Fluorophore-conjugated streptavidin was used to assess the biotinylation activity of the MyoJ-TurboID-3Ty fusion. In absence of biotin addition to the medium, the main signal of biotinylated proteins was visible at the apicoplast with a weaker signal for the mitochondrion and the posterior pole of intracellular parasites (**Figures 1A, B**). As previously reported, the background at the apicoplast and mitochondrion results from metabolic enzymes using biotin as a co-factor, such as carboxylases (Jelenska et al., 2001), whereas the weak signal at the posterior pole indicates a slight leakiness of the TurboID. In contrast, when biotin was added to the medium for 7 h on intracellular parasites (**Figure 1A**) or 4 h on extracellular parasites (**Figure 1B**), a strong signal was observed at the basal pole while the signal of the apicoplast and mitochondrion, conversely, appeared weaker. These observations indicate that MyoJ-TurboID-3Ty fusion is active and able to efficiently biotinylate proteins at the basal complex within a period of a few hours.

To identify resident components and possibly transient interacting proteins of the basal complex, the biotin labeling was performed over 7 h on HFF cells highly infected with MyoJ-TurboID-3Ty or the parental ΔKU80 strain, starting a few hours before tachyzoite egress and stopping once almost all the parasites were released from the host cells. Streptavidin affinity pull-down experiments were performed in duplicate for the MyoJ-TurboID-3Ty cell line using ΔKU80 strain as control (**Figure 1C**). A strong enrichment of biotinylated proteins was observed in the case of MyoJ-TurboID-3Ty, and the samples were further analyzed by mass spectrometry. All the detected proteins are listed in **Supplementary Table 3**. We focused our analysis on the biotin-treated samples and, more especially, on proteins having an abundance ratio strictly greater than 2 compared to the control or detected in the MyoJ-TurboID-3Ty samples but not in the control (log2(MyoJ+biotin/ΔKU80+biotin)>1 in **Figure 1D**). 114 proteins were thus significantly enriched and common in the two replicates, including most of the known basal complex proteins, namely, MORN1, CEN2, IMC5, IMC8, IMC9, and IMC13 (Gubbels et al., 2006; Hu, 2008; Anderson-White et al., 2011) as well as IMC proteins previously reported as nearby, SIP/CBAP, GAPM2B, IMC6, IMC10 and IMC14 (Bullen et al., 2009; Anderson-White et al., 2011; Tilley et al., 2014; Lentini et al., 2015) (**Figure 1D**).

12 candidates, listed in **Table 1**, were selected for further analysis based on their enrichment, their similarity with proteome localization screen data, and/or expression pattern of known basal pole proteins (Behnke et al., 2010; Barylyuk et al., 2020) (**Supplementary Figure 2A**). Recently, eight of our candidates were identified by a multi-bait proximity biotinylation approach and named BCCs for Basal Complex Components, so we kept here the same nomenclature for consistency (Engelberg et al., 2022) (**Table 1**). Among our 12 candidates, two are kinases, TGGT1_21872 (CDPK6) and TGGT1_231070 (BCC2), and two are phosphatases, predicted to encode a Protein Phosphatase 2A (PP2A) homolog domain (TGGT1_269460, BCC5) and an NLI-interacting factor-like phosphatase domain (TGGT1_202550, BCC6) according to SMART (Letunic et al., 2021) (**Supplementary Figure 2B**). These enzymes could regulate basal pole activities since phosphorylation sites have been identified in most of the known basal complex proteins (Treeck et al., 2011). The eight remaining proteins were annotated as hypothetical proteins. No functional domains were predicted for TGGT1_215380 (Candidate 1 or C1), TGGT1_209420 (C2) and TGGT1_273050 (BCC8) while the bioinformatic analysis detected an armadillo (ARM) repeat for TGGT1_273905 (C3), short coiled-coils for TGGT1_232780 (BCC1), TGGT1_311230 (BCC7), and TGGT1_278130 (BCC11), and two guanylate-binding protein domains for TGGT1_283510 (BCC10) (**Supplementary Figure 2B**). According to the genome-wide CRISPR screen performed in the tachyzoite (Sidik et al., 2018), none of these proteins are predicted to be essential for parasite growth, and only BCC2 and C3 are conserved throughout the apicomplexan phylum when analyzed by OrthoFinder (Emms and Kelly, 2019) (**Supplementary Table 4**). The other BCCs are mainly found in the cyst-forming subgroup of coccidian parasites except CDPK6, also present in *Plasmodium* species.

**Table 1:** Summary of the information about the 12 candidates investigated in this study.

TGGT1 gene id.	Name	ToxoDB description; SMART/PFAM description	Fitness score	Nb of phos. sites; Predicted palm. cyst	Localization/function(this study)	References
215380	C1	hypothetical protein	1,92	10	Cytosol/n.d.	
209420	C2	hypothetical protein	-2,96	9	Cytosol/n.d.	
273905	C3	hypothetical protein; ARM repeat	-0,53	5	Cytoplasm/n.d.	
218720	CDPK6	calcium-dependent protein kinase 6; S/T protein kinase, 6 EF-hand domains	-0,01	0	Posterior cup/ no phenotype	(Long et al., 2016)
232780	BCC1	hypothetical protein; Coiled-coil	-1,88	4	Posterior cup/constriction	(Engelberg et al., 2022)
231070	BCC2	protein kinase S/T protein kinase, catalytic domain	0,22	29 C313-314-828-1140	Above the basal ring/no phenotype	(Engelberg et al., 2022)
269460	BCC5	Ser/Thr phosphatase family protein; Protein phosphatase 2Ac homolog, 1 coiled-coil	-0,81	36 C886-887	Posterior cup/ connection	(Engelberg et al., 2022)
202550	BCC6	NLI-interacting factor (NIF) family phosphatase; 2 NIF domains, 1 coiled-coil	-0,39	34 C739-747-748-1469	Above the basal ring/no phenotype	(Engelberg et al., 2022)
311230	BCC7	hypothetical protein; TMD, Apolipophorin III domain, 4 coiled-coils	0,74	129 C22-2826	Above the basal ring/no phenotype	(Vigetti et al., 2022; Engelberg et al., 2022)
273050	BCC8	hypothetical protein	2,08	0	Below the posterior cup/connection	(Engelberg et al., 2022)
310220	BCC10	hypothetical protein; Guanylate-binding protein domain	-1,36	28 C910	Below the posterior cup/connection	(Engelberg et al., 2022)
278130	BCC11	hypothetical protein; 2 internal repeats, 3 coiled-coils	-0,1	16	Below the posterior cup/connection	(Engelberg et al., 2022)

Id.: identifier, fitness score according to the genome-wide CRISPR screen (Sidik et al., 2018), Number of phosphorylation sites according to the tachyzoite phosphoproteome from purified parasite or infected host cell [RH, (Treeck et al., 2011)], palmitoylated cysteines have been predicted using CSS-Palm 4.0 (http://csspalm.biocuckoo.org/online.php) with a high threshold. The localization and function of proteins are those described in this study. n.d: not determined, cyst or C: cysteine, S/T: serine/threonine.

### Chronological association of the identified proteins with the basal complex

**Figure 2:**
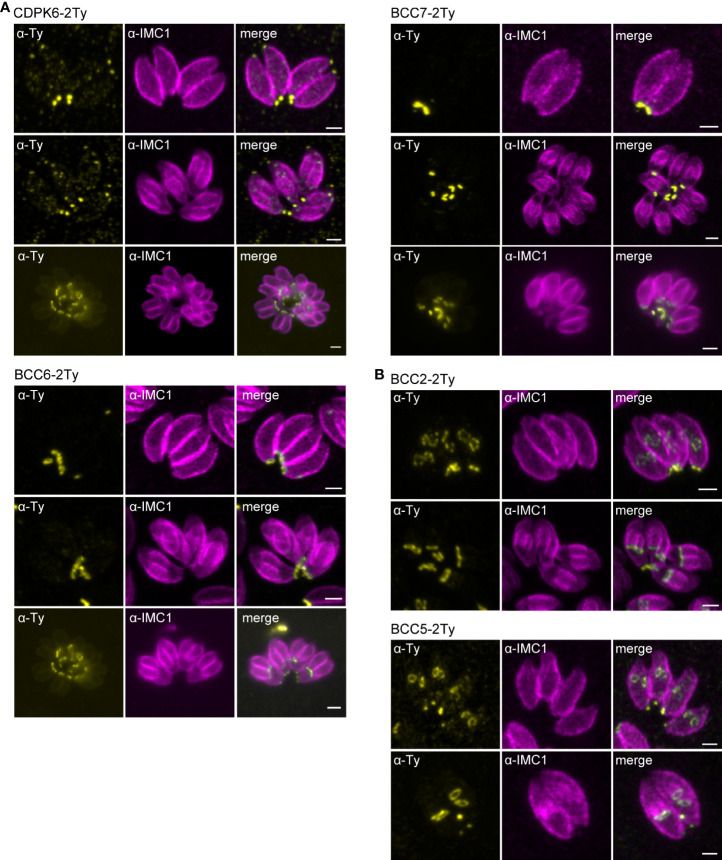
BCCs targeted to the basal complex very late or very early during cell division. **(A)** Endogenous tagging of CDPK6, BCC6, and BCC7 shows that these proteins appear at the basal complex in mature parasites only. Note that CDPK6 is also found at the apical pole of the parasites including in the daughter cells. **(B)** In contrast, BCC2 and BCC5 are observed very early during division, appearing as a ring before the IMC1 staining of the daughter cells. Magenta: rabbit anti-IMC1, Yellow: mouse anti-Ty. Scale bars: 2 μm.

**Figure 3:**
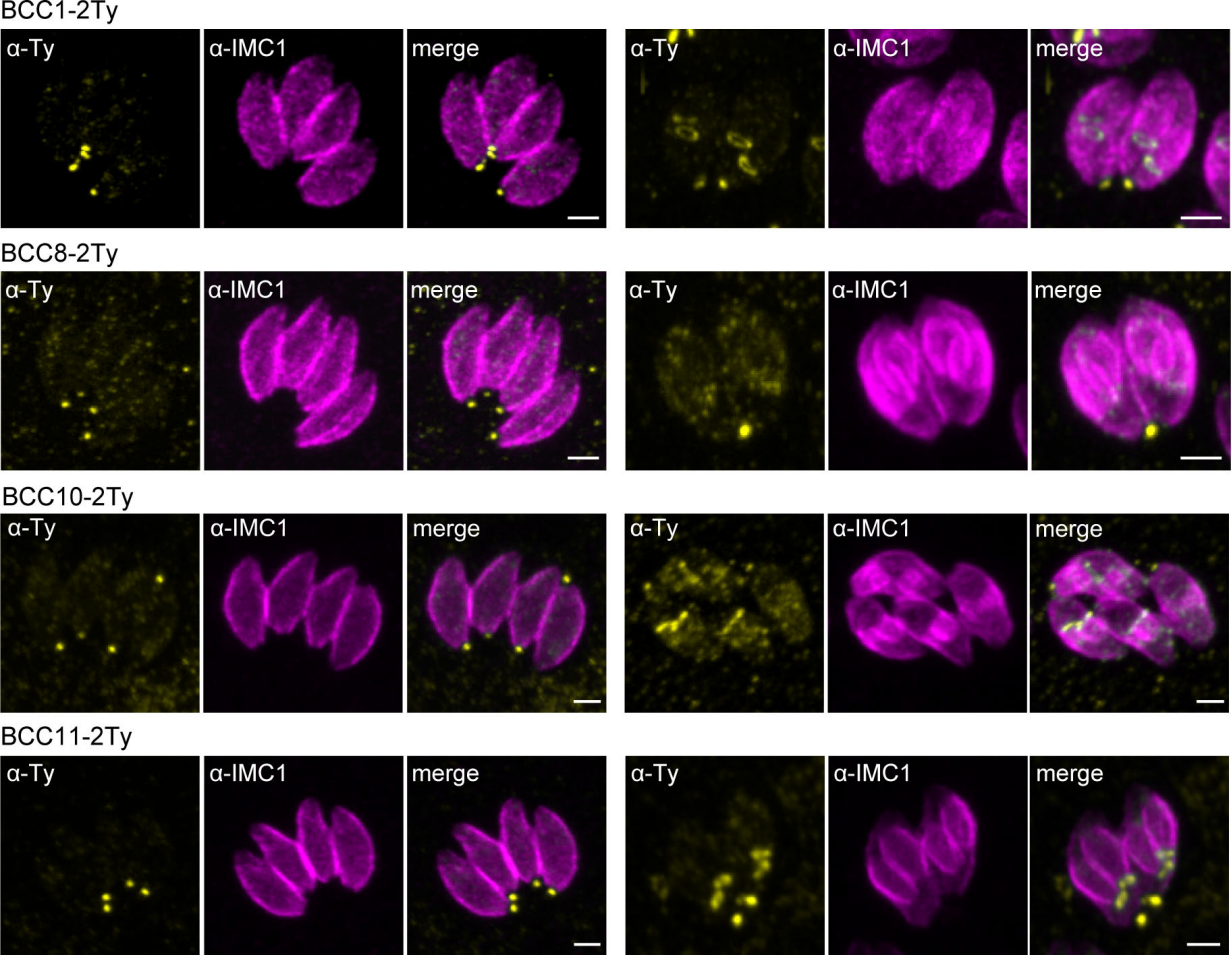
BCCs present to the basal complex all along the IMC elongation. Immunofluorescence assays performed on parasites expressing the endogenously tagged version of BCC1, BCC8, BCC10, and BCC11 show that all these BCCs cap the basal end of the IMC during the development of the daughter cells and in the mature parasites. Magenta: rabbit anti-IMC1, Yellow: mouse anti-Ty. Scale bars: 2 μm.

To map their subcellular localization within the tachyzoite, the 12 candidates were endogenously Ty-tagged at their C-terminus. Three of them, candidates 1-3 (C1-3), displayed a punctate signal in the cytosol (C1 and C2) or cytoplasm (C3) (**Supplementary Figure 2C**; **Table 1**). The nine other candidates were found at the basal complex but associated with this structure with selective kinetics during division. Firstly, as with the recently characterized BCC7 (Vigetti et al., 2022), CDPK6 and BCC6 were observed only in the mature parasites but not in the developing daughter cells except when they are very nearly ready to emerge from the mother cell (**Figure 2A**). The staining of CDPK6 showed a dot-like signal distant from the basal end of the IMC, suggesting a localization at the basal cup of the parasites. CDPK6 is additionally observed at the apical tip of the tachyzoites, including in the growing daughter cells. In contrast, BCC6, like BCC7, was detected as a ring closer to the basal edge of the IMC, similarly to what has been reported for MyoC-glideosome complex (Frénal et al., 2014). Secondly, BCC2 and BCC5 were found in the basal complex of both the growing daughter cells and mature parasites and were organized as rings very early during the cell division (**Figure 2B**). Indeed, BCC2 and BCC5 were detected before IMC1 in the daughter cells. Finally, all the other BCCs were observed in the basal complex of mature parasites and developing daughter cells but never before the emergence of the IMC1 staining (**Figure 3**). Similarly to MyoJ, BCC1, BCC8, BCC10, and BCC11 appeared as basal dots distant from the end of the IMC in mature parasites and as rings capping the basal edge of the IMC in growing progenies (**Figure 3**). Of note, the staining of BCC8 was less intense, particularly in the developing daughter cells.

Taken together, the spatial proteomics analysis identified nine basal complex components and highlighted a hierarchy of expression during the assembly and development of the daughter cell cytoskeleton.

### Mapping of the BCCs revealed two new sub-compartments of the basal complex

**Figure 4:**
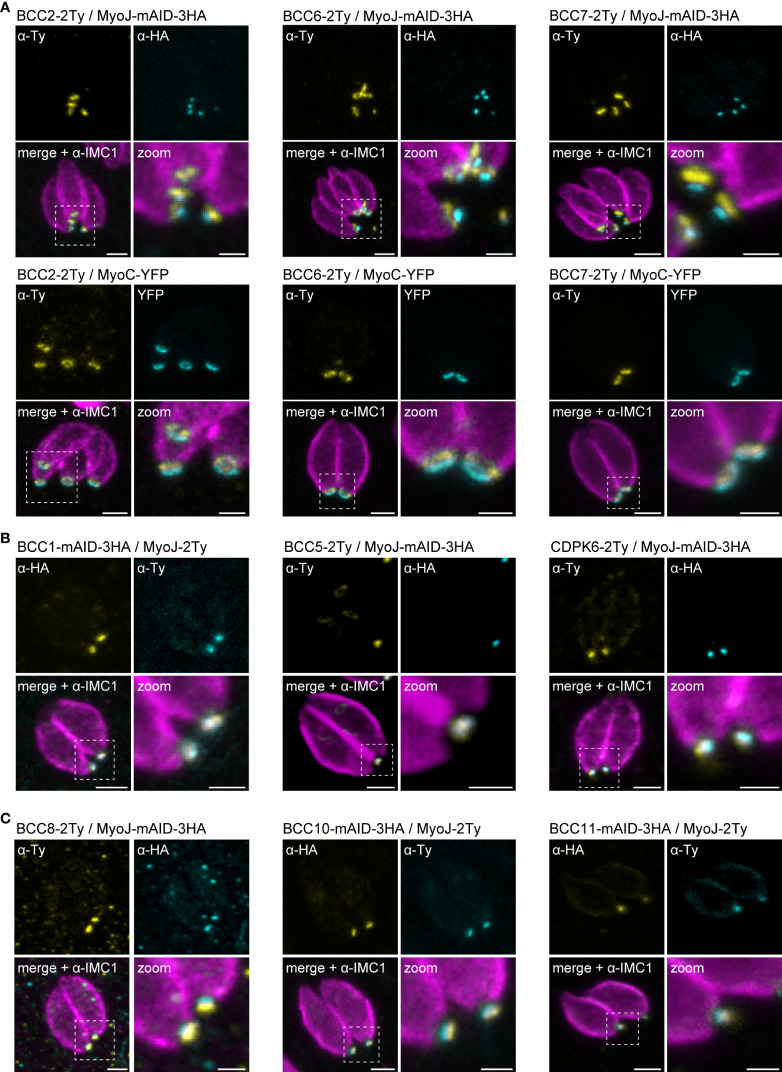
Mapping of the BCCs in three sub-compartments of the basal complex, including two new ones. **(A)** Co-localization of BCC2, BCC6, and BCC7 with endogenously tagged MyoJ and MyoC showing that these three proteins delineate a new sub-compartment located between the basal end of the IMC and the basal ring. **(B)** BCC1, BCC5, and CDPK6 co-localize with endogenously tagged MyoJ at the posterior cup. **(C)** A comparison of BCC8, BCC10, and BCC11 staining with endogenously tagged MyoJ shows that these three proteins are located below the posterior cup. Magenta: IMC1, Yellow: BCC of interest, Cyan: MyoC or MyoJ. Scale bars: vacuoles, 2 μm; zooms, 1 μm.

Based on the co-staining with IMC1, the localization of the BCCs resembled those of MyoC or MyoJ, delineating the basal ring and the posterior cup, respectively. To validate these first observations, additional co-localization experiments were performed with endogenously tagged MyoC (MyoC-YFP) or MyoJ (MyoJ-mAID-3HA). This revealed that, like BCC7, BCC2 and BCC6 are distributed above the basal cup but also above the basal ring, in a position that likely matches with the recently reported new sub-compartment of the basal complex (**Figure 4A**) (Vigetti et al., 2022). In contrast, BCC1, BCC5, and CDPK6 showed an overlap with MyoJ and, therefore, likely reside at the level of the posterior cup (**Figure 4B**). Finally, and somehow surprising, BCC8, BCC10, and BCC11 were observed under the signal of MyoJ (**Figure 4C**), thus below the basal cup described so far as the most basal sub-compartment of the basal complex (Gubbels et al., 2022).

**Figure 5:**
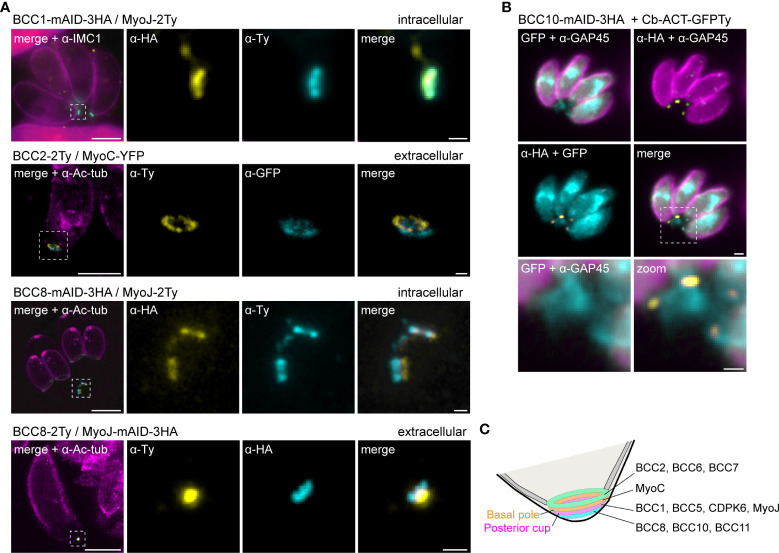
The basal complex is organized in four ring-shaped structures. **(A)** Co-staining of BCC1, BCC2, and BCC8 with endogenously tagged MyoJ or MyoC confirms their localization into the basal complex. BCC1 co-localizes with MyoJ at the posterior cup, while and BCC2 is located in a new sub-compartment above the basal ring (MyoC staining). Finally, co-labeling of BCC8 in a MyoJ-tagged strain corroborates the presence of a ring structure under the basal posterior cup that appears highly constricted in extracellular parasites compared to intracellular. Magenta: IMC1 or acetylated tubulin (Ac-tub), Yellow: BCC of interest, Cyan: MyoC or MyoJ. Scale bars: vacuole, 10 μm; zooms, 1 μm. **(B)** Co-localization of BCC10 within intravacuolar connections marked by F-actin chromobodies (Cb-ACT-GFPTy) and parasite periphery marked by GAP45. Magenta: GAP45, Yellow: BCC10, Cyan: F-actin. Scale bars: vacuole, 2 μm; zooms, 1 μm. **(C)** Model of the basal pole of the tachyzoite with the location of the BCCs. According to the mapping performed in this study, the basal complex is organized in at least four superimposed ring-shaped sub-compartments. The previously described basal ring and posterior cup are orange and pink, respectively.

To further resolve the basal complex organization and structure, we had a closer look at some of these BCCs using ultrastructure expansion microscopy (U-ExM), which allowed a four-fold expansion of the samples and a corresponding gain in resolution (Dos Santos Pacheco and Soldati-Favre, 2021; Gambarotto et al., 2021). At this scale, BCC1 was detected as a ring that co-localized with MyoJ, in line with a basal ring position (**Figure 5A**). BCC2 was detected as a large ring above the basal ring defined by MyoC. Finally, BCC8 was also found as a ring in intracellular parasites below the posterior cup and sometimes overlapping with it. In extracellular parasites, this signal appeared more constricted and formed a dot under MyoJ.

BCC8, BCC10, and BCC11 signals were distant from the edges of the IMC, so to evaluate their location with respect to the connection and residual body, chromobodies recognizing specifically filamentous actin (Cb-ACT-GFPTy) were transiently expressed in the BCC8-tagged cell line. These chromobodies revealed an extensive filamentous network located mainly in the cytoplasmic bridges that connect the progenies within their parasitophorous vacuole and also in a juxtanuclear region (Periz et al., 2017; Tosetti et al., 2019). We observed that Cb-ACT and BCC8 fluorescent signals overlapped, and we hypothesized that the lower sub-compartment of the basal complex is at the border between the parasite and the connection linking them (**Figure 5B**).

Overall, the detailed mapping of the identified BCCs with respect to established markers of the basal ring and posterior cup unveils the existence of four superimposed ring-shaped structures within the basal complex composed, from the edge of the IMC to the bottom of the parasite, of (i) BCC2, BCC6 and BCC7, (ii) MyoC-glideosome complex, (iii) MyoJ, BCC1, BCC5, and CDPK6, and (iv) BCC8, BCC10 and BCC11 (**Figure 5C**).

### BCC1 functions with MyoJ to constrict the basal complex of the tachyzoite

To assess the function of the BCCs during the tachyzoite lytic cycle, an auxin-inducible knock-down cell line was generated for each protein in a MyoJ-tagged background (MyoJ-2Ty) that provided the opportunity to track the fate of the basal pole. For every BCC tested except BCC1, we neither detected a change in the MyoJ-2Ty localization nor a defect in basal pole constriction (**Supplementary Figure 3**).

**Figure 6:**
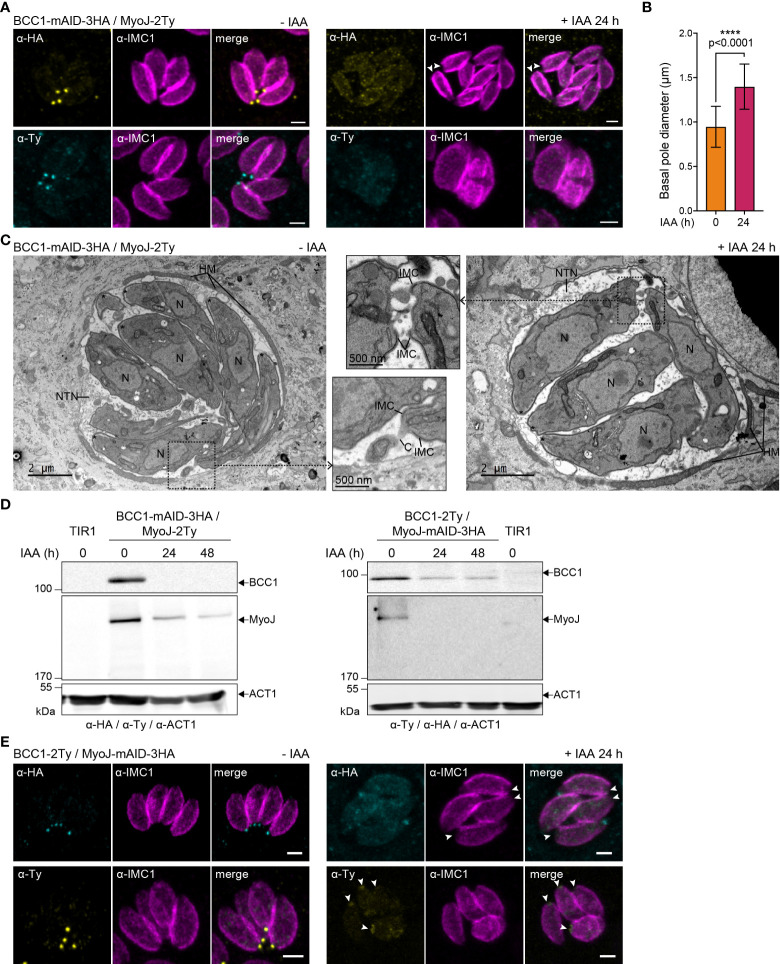
BCC1 is involved in the constriction of the basal complex. **(A)** Localization of endogenously tagged MyoJ (MyoJ-2Ty) in the auxin-inducible knockdown of BCC1 (BCC1-mAID-3HA) assessed by immunofluorescence (IFA) after a 24 h treatment with or without 500 μM of auxin (IAA). Magenta: IMC1, Yellow: BCC1, Cyan: MyoJ. Scale bars: 2 μm. **(B)** Diameter of the basal pole of BCC1-mAID-3HA/MyoJ-2Ty tachyzoites ± IAA, measured on IFA pictures using the IMC1 staining. The distance between the basal edges of the IMC has been measured in 53 non-treated and 108 treated parasites. The results are represented as mean ± SD, and their significance was assessed using an unpaired t-test, with the two-tailed p-value written on the graph. **(C)** Electron microscopy pictures of BCC1-mAID-3HA cell line non-treated and treated with IAA for 24 h. On the panel in the middle, zoom on the basal pole. Asterisks show the parasites apical pole. C, intracellular connection; HM, host mitochondria; N, nucleus; NTN, nanotubular network. **(D)** The expression of BCC1-2Ty and MyoJ-2Ty was assessed in the MyoJ-mAID-3HA and BCC1-mAID-3HA cell lines, respectively, after 0, 24, and 48 h of IAA treatment. **(E)** Localization of endogenously tagged BCC1 (BCC1-2Ty) in the auxin-inducible knockdown of MyoJ (MyoJ-mAID-3HA) assessed by IF after a 24 h treatment with or without 500 μM of IAA. Magenta: IMC1, Yellow: BCC1, Cyan: MyoJ. Scale bars: 2 μm.

In contrast, depletion of BCC1 led to a loss of (i) rosette organization, (ii) synchronicity of division, and (iii) MyoJ staining at the basal cup (**Figure 6A**). Observation of the IMC1 signal in the area of the basal pole also suggested a loss of constriction. Thus, the distance between the basal edges of the IMC was measured. In BCC1-depleted parasites, the basal pole diameter is found to be 1,5 time larger than in untreated cells (0,95 μm ± 0,23 vs 1,40 μm ± 0,25, **Figure 6B**). Electron microscopy further confirmed the constriction defect of the parasites after 24 h of auxin (IAA) treatment but showed that the parasite ultrastructure, capacity to form the typical nanotubular network in the parasitophorous vacuole and to recruit host cell mitochondria were preserved (**Figure 6C**). Western blot analysis of the strain also showed that depletion of BCC1 strongly affected the stability of MyoJ (**Figure 6D**). Consequently, to assess if the localization and expression of BCC1 were also impacted by the absence of MyoJ, BCC1 was conversely tagged in an auxin-inducible cell line of MyoJ (MyoJ-mAID-3HA, **Supplementary Figure S4A**) that, in presence of auxin, reproduces the defect of basal pole constriction and intravacuolar parasite connection reported for the MyoJ-KO strain (Frénal et al., 2017) (**Supplementary Figure S4B**). We did observe that BCC1 staining at the basal pole was weaker and even undetectable in more than 50% of the parasites when MyoJ was depleted (**Figure 6E**). The stability of BCC1 was indeed highly reduced in this condition, as shown by western blot (**Figure 6D**). These results strongly suggest that BCC1 and MyoJ are part of the same complex, which contributes to the constriction process of the parasite basal pole.

**Figure 7:**
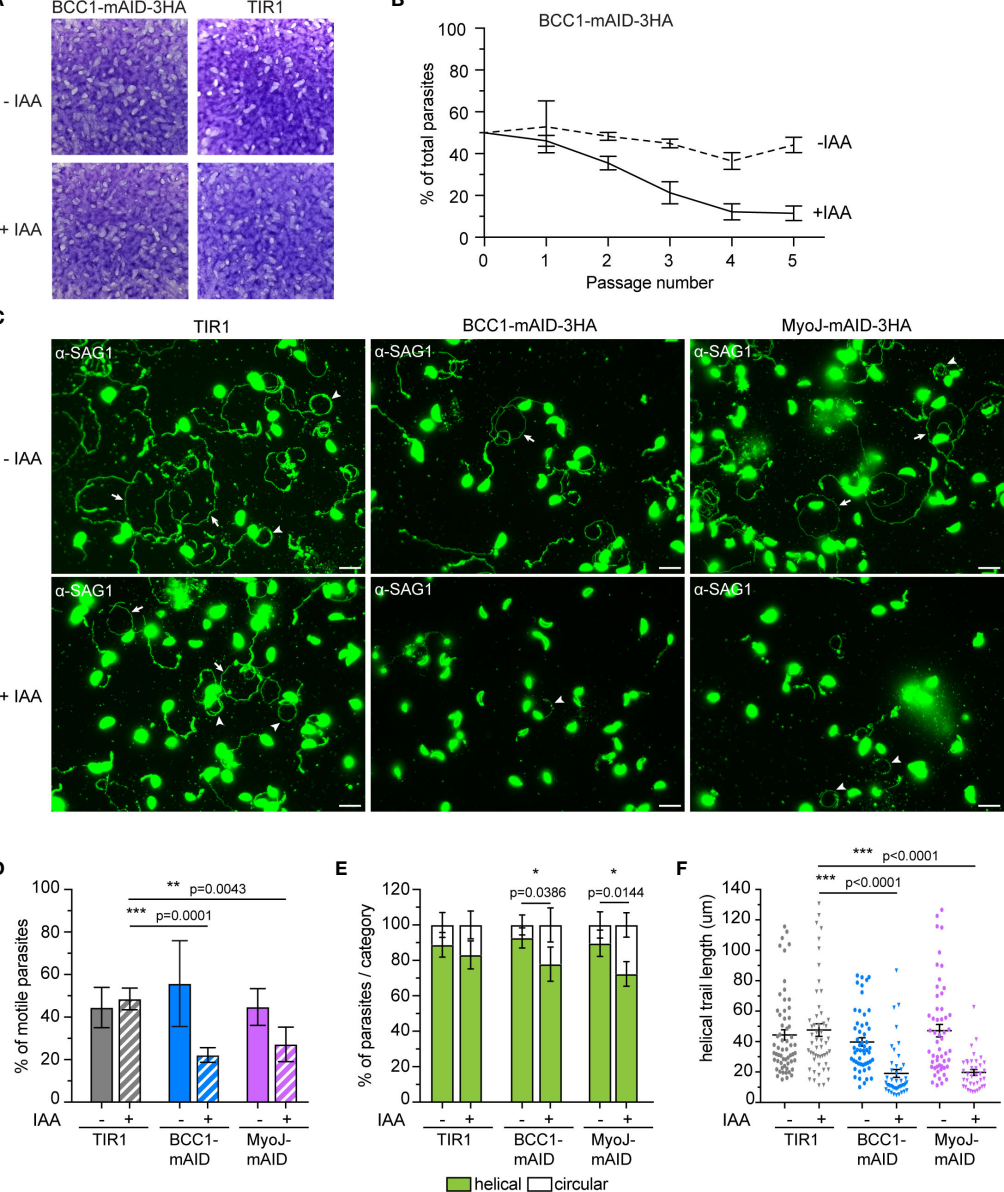
BCC1 depletion impairs parasite fitness and motility *in vitro.* **(A)** Plaque assays performed with BCC1-mAID-3HA/MyoJ-2Ty and the parental TIR1 strains over 7 days treated or not with auxin (IAA). **(B)** Competition assay performed over 5 passages (14 days) with BCC1-mAID-3HA/MyoJ-2Ty treated or not with IAA. The experiment has been done in triplicate, and the results are presented as mean ± SD. **(C)** Trail deposition staining after gliding of control (TIR1), BCC1-mAID-3HA/MyoJ-2Ty, AND MyoJ-mAID-3HA/BCC1-2Ty parasites treated or not for 24 h with 500 μM of IAA. Parasites were allowed to glide on poly-L-lysine-coated coverslips for 10 min in saline buffer, and trails were stained with anti-SAG1 (green) Scale bars: 10 μm. The arrows point towards helical gliding while arrowheads point to circular gliding trails. **(D)** Quantification of the overall parasite motility from trail deposition assays. The total number of trails (circular, helical and short) and parasites were determined for 6 to 10 fields per sample, in duplicates, and for four independent experiments. **(E)** Quantification of the types of motility performed by the parasites from the same fields and categorized in two groups, helical or circular. **(F)** Measure of the length of the helical trails on the same fields. For **(D, E)**, the results are presented as mean ± SD; for **(F)** the results are presented as mean ± SEM. Their significance was assessed using an unpaired t-test with the two-tailed p-value written on the graphs. * for p<0.05, ** for p<0.01, *** for p<0.001.

As previously reported for MyoJ-KO parasites (Frénal et al., 2017), no defect was observed during the lytic cycle of the tachyzoites lacking BCC1 (**Figure 7A**), although these parasites showed a slight and cryptic reduction in growth fitness compared to untreated cells in a competition assay (**Figure 7B**). To pinpoint the defect(s) of these BCC1-deficient parasites, we sought to investigate motility based on previous reports that have identified the parasite basal pole as a periodic adhesion site required to generate the forces driving both *T. gondii* tachyzoite and *P. berghei* sporozoite motility (Münter et al., 2009; Pavlou et al., 2020). In addition, it was reported that the tension provided by the cytoskeleton and the shape of the parasite, in particular curvature, likely direct the gliding pattern (Pavlou et al., 2020; Hueschen et al., 2022). Therefore, we hypothesized that the non-constricted basal pole of the BCC1-KD and MyoJ-KD parasites could impede their gliding. Motility assays were performed on poly-L-lysine coated coverslips to visualize the trails deposited by freshly egressed parasites. For the parental strain (TIR1) and for parasites expressing BCC1- or MyoJ-mAID-3HA, we observed long helicoidal trails and circles corresponding to helical and circular gliding, respectively (**Figure 7C**). However, the addition of auxin significantly decreases the ability of the BCC1- and MyoJ-depleted parasites to glide compared to the TIR1 strain (**Figure 7D**), as shown by the overall reduced number of trails observed (**Figure 7C**). Quantification of the trajectories of the motile parasites showed that the treated parasites produced a little less helical movements, a movement known to efficiently propel the parasite forward, compared to the untreated ones (**Figure 7E**). In addition, the length of the helical trails was significantly shorter for the BCC1- and MyoJ-depleted parasites, indicating that these parasites glide on shorter distances (**Figure 7F**).

To observe gliding in a more physiological condition, natural egress and subsequent gliding motility were followed by live microscopy with the BCC1-mAID-3HA strain (**Supplementary Movies 1, 2**). We first noticed that most of the auxin-treated parasites exited the host cell at the same speed as the untreated ones, but some were delayed. We also observed that these parasites moved for a shorter time and that their movements were often jerky. This was especially the case for the stationary twirling when the parasites stand up vertically and spin on their posterior pole.

Overall, these results indicate that BCC1 and MyoJ are not critical for tachyzoite growth but are required for constricting the basal pole at the end of the cell division process. The loss of BCC1 and MyoJ impacts the shape of the progenies and the intracellular rosette organization. The mild fitness defect observed during the competition assay likely results from alteration of their motile skills.

### BCC5, BCC8, BCC10, and BCC11 are involved in the formation and/or maintenance of the intravacuolar connection

**Figure 8:**
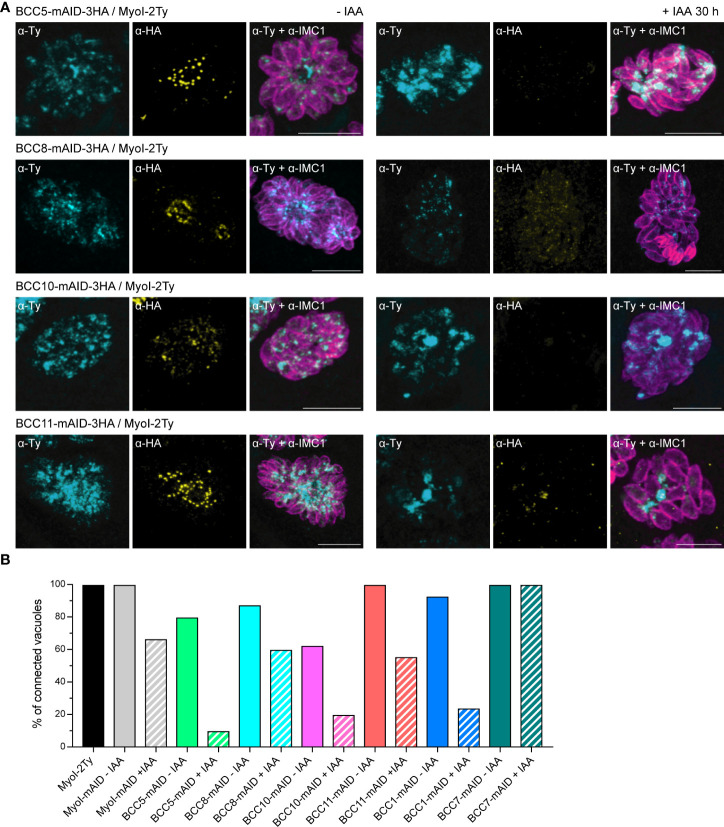
BCC5, BCC8, BCC10, and BCC11 depletion impacts the rosette organization of the parasites and their intravacuolar connection. **(A)** Immuno-detection of MyoI endogenously tagged (MyoI-2Ty) in an auxin-inducible knockdown of BCC5, BCC8, BCC10, and BCC11 assessed by IFA after 30 h treatment with or without 500μM of IAA. Magenta: IMC1, Yellow: BCC of interest, Cyan: MyoI. Scale bars: 10μm. **(B)** Percentage of vacuoles in which the parasites were connected to each other’s. This was assessed by FRAP experiment using the soluble GFP as a reporter of diffusion. See **Supplementary Figure 5** for the significance of the results (representative vacuoles for “connected” and “non-connected” as well as for the number of bleached vacuoles).

As mentioned previously, depletion of the other BCCs had no effect on the localization of MyoJ and constriction of the basal complex. However, we noticed that individual depletion of BCC5, BCC8, BCC10, and BCC11 altered the rosette organization of the parasites and their intravacuolar synchronicity of division without impacting other aspects of the lytic cycle (**Supplementary Figure 5A**). Of note, the tagging of BCC10 with the mAID domain already impaired the rosette organization without adding auxin to the medium, suggesting a mild phenotype induced by reduced protein function. This phenotype is reminiscent of the MyoI-KO. This myosin heavy chain has been localized mainly in the cytoplasmic bridges between the parasites and shown to be critical for their formation and/or maintenance (Frénal et al., 2017). We, therefore, assayed MyoI localization in the background of auxin-inducible KD strains of BCC5, BCC8, BCC10, and BCC11 by endogenous tagging (**Figure 8A**). In non-treated parasites, MyoI staining was mainly observed at the center of the vacuole, where the parasites are connected to the residual body except for the BCC10 cell line, in which the signal was more homogeneously distributed. In contrast, in the presence of auxin, a substantial accumulation of MyoI inside the growing BCC5-KD was noticed, while the signal of MyoI was less intense in the parasites depleted in BCC8, BCC10, and BCC11.

Because these results suggested a loss of intravacuolar connection between parasites, we performed fluorescence recovery after photobleaching (FRAP) experiments as previously described for MyoI-KO (Frénal et al., 2017). The BCC5-, BCC8-, BCC10-, and BCC11-mAID-3HA strains were transiently transfected with the soluble protein GFP and subsequently treated or not with auxin for 28-30 h. We then evaluated the capacity of the GFP protein to diffuse between parasites, sharing the same parasitophorous vacuole. This was achieved by photobleaching at least one parasite in vacuoles containing 2, 4, or 8 parasites and recording the fluorescence intensity over time in all the parasites of the vacuole (**Supplementary Figure 5B**). Using MyoI-2Ty and MyoI-mAID as control strains, we found a significant decrease in the GFP diffusion in the BCC5-, BCC8-, BCC10-, and BCC11-depleted parasites (**Figure 8B**). The same result was observed for BCC1-KD parasites, used as a positive control, whereas the BCC7-mAID strain, used as a negative control, showed no difference upon auxin treatment.

Taken together, these results identify BCC5, BCC8, BCC10, and BCC11 as direct contributors to the rosette organization of intracellular tachyzoites through the formation and/or the maintenance of the connection between the progenies.

## Discussion

The basal complex is a cytoskeletal structure appearing very early during the emergence of the daughter cells and capping the newly formed IMC along its elongation within the cytoplasm of the mother parasite. Therefore, to avoid cytoplasmic contamination as much as possible, we choose to use the TurboID, which allows faster biotinylation as compared to the BirA (Branon et al., 2018), and to induce biotinylation of proteins during the last hours of the tachyzoite lytic cycle, prior, during and after their egress from the infected host cells. The mass spectrometry data demonstrate that the TurboID robustly mapped proteins adjacent to MyoJ in a few hours. Indeed, most of the known basal complex proteins were significantly enriched in the samples, including MORN1, CEN2, IMC5, IMC8, IMC9, and IMC13 (Gubbels et al., 2006; Hu, 2008; Anderson-White et al., 2011). Several others were also found but fell under our set threshold (abundance ratio >2), including MyoC and its anchoring protein IAP1 (IMC-associated protein 1) (Frénal et al., 2014), MSC1 (Mature Soluble Cytoskeleton protein 1) and 14-3-3 (Lorestani et al., 2012) (**Supplementary Table 3**). Several IMC-associated or IMC-embedded proteins were also enriched in the samples, probably because of their close proximity with the basal complex, such as several alveolin repeat-containing proteins (Anderson-White et al., 2011), the five glideosome-associated proteins with multiple-membrane spans [GAPM, (Bullen et al., 2009)], and two proteins found in the transverse sutures of the IMC that cap the basal complex, namely the protein S-acyl transferase DHHC14 (Dogga and Frénal, 2020) and CBAP/SIP (Tilley et al., 2014; Lentini et al., 2015). However, other known proteins have not been detected, such as the gliding-associated protein GAP80 (Frénal et al., 2014), the phosphatase HAD2a (Engelberg et al., 2016), the kinase FIKK (Skariah et al., 2016), the *Chlamydomonas reinhardtii* deflagellation inducible protein homolog SSNA1/DIP13 (Lévêque et al., 2016) or the myosin heavy chain MyoI (Frénal et al., 2017).

We shortlisted 12 putative basal complex proteins taking into account their significant enrichment in both samples and comparing their putative localization and cell cycle profile with the known basal pole proteins (Behnke et al., 2010; Barylyuk et al., 2020). We confirmed the posterior localization for nine of them by endogenous tagging, the three remaining ones being found in the cytoplasm of the parasites. Our study implements and strengthens the results recently presented in a multi-bait proximity biotinylation approach using the basal proteins MORN1, IMC8, and CEN2 as fusion proteins for the biotin ligase BirA (Engelberg et al., 2022). Indeed, although most of our candidates have been identified, their detailed localization within the basal pole and their molecular function were not addressed, except for BCC1. Only CDPK6 was not localized in this study, although the authors suggested its basal location. We confirm herein such position by localizing this protein in the posterior cup together with MyoJ. Moreover, the authors found four other BCCs that we have not investigated here: BCC3 (TGGT1_311770) and BCC4 (TGGT1_229260), because they were under our threshold, and BCC0 (TGGT1_294860) and BCC9 (TGGT1_200330), because they have not been identified in our mass spectrometry analysis. This discrepancy is likely due to the absence of BCC0, BCC3, and BCC4 in mature parasites since our experimental set-up focused on the last hours of the lytic cycle. In contrast, two heat shock proteins, HSP21 and HSP29, were found significantly enriched in the two studies and would certainly deserve further investigations.

To get a deeper understanding of the basal complex’s assembly mechanism, we followed the recruitment of the BCCs during cell division using IMC1 as a marker of the mature and developing parasites. During endodyogeny, BCC2 and BCC5 were observed first, appearing as a ring-shaped structure preceding the detection of IMC1 in the progenies. Since IMC1 is absent from the apical cap, this suggests that BCC2 and BCC5 are expressed before or concomitantly to the formation of the apical cap. Then, BCC1, BCC8, BCC10, and BCC11, similarly to MyoJ, were additionally found at the basal complex, capping the basal edges of the daughter cell IMC throughout its elongation. Finally, CDPK6, BCC6, and BCC7 are recruited to the basal complex at the end of the cell division, when the daughter cells are almost ready to bud from the mother. All the BCCs remain visible in the mature parasites and throughout the successive division rounds.

To determine the organization of the BCCs within the basal complex, we then performed a detailed mapping using markers of its two known sub-compartments, MyoC for the basal ring and MyoJ for the posterior cup, and benefiting from super-resolution microscopy (U-ExM) (Gambarotto et al., 2021). We observed that several BCCs did not co-localize with neither MyoC nor MyoJ. This is the case for the phosphatase BCC6 and the kinase BCC2, which were found to form rings above that of MyoC, sometimes slightly overlapping it, as recently reported for BCC7 (Vigetti et al., 2022). These results indicate the presence of an upper sub-compartment that might link the basal complex to the overlying IMC (**Figure 5C**). Supporting this hypothesis, co-immunoprecipitation assays performed with the very large BCC7 protein (>550 kDa) pulled down five known IMC proteins and two new ones (Vigetti et al., 2022). Surprisingly, no BCCs have been co-localized with MyoC at the basal ring, and none of the MyoC-glideosome components have been significantly enriched in the mass spectrometry data. However, we localized three BCCs, BCC1, the phosphatase BCC5, and the kinase CDPK6, at the posterior cup, overlapping with the MyoJ signal and three others, BCC8, BCC11, and the guanylate-binding protein BCC10, below, defining an additional sub-compartment at the border between the posterior cup and the nanotube connecting each parasite of the same vacuole to the residual body. This latter compartment appears as a ring in intracellular parasites and is highly constricted in extracellular parasites, being visualized as a dot by U-ExM.

Finally, we undertook a functional analysis of the BCCs, using the auxin-inducible degron system fused to the 3’ end of the genes of interest. The addition of auxin successfully depleted the BCCs to undetectable levels. None of them showed a striking growth defect for the tachyzoites *in vitro*, confirming the fitness score measured in the CRISPR-Cas9-based genome-wide genetic screen of *T. gondii* (Sidik et al., 2016). However, observing the generated cell lines, two phenotypes could be noted. First, in BCC1-KD parasites, the typical rosette organization was lost, an asynchrony of division within the vacuoles was observed, and the basal pole was significantly enlarged. This phenotype was reminiscent of MyoJ-KO parasites (Frénal et al., 2017). Therefore, the expression and localization of MyoJ were followed during the depletion of BCC1, and conversely, BCC1 was observed upon MyoJ down-regulation. These experiments established that depletion of one causes the destabilization of the other and *vice-versa*, suggesting an interaction between those two proteins. Because of their phenotype and co-localization at the posterior cup, we propose that BCC1 and MyoJ are parts of the contractile ring machinery of the tachyzoite. BCC1 has no predicted structural or functional domain except a short coiled-coil region that might allow self-dimerization of the protein. Upon depletion of BCC1, we never observed MyoJ at the basal pole, while we did observe some residual staining of BCC1 in MyoJ-depleted parasites. This suggests that BCC1 might be a cargo for MyoJ interacting in the tail region of this myosin heavy chain. Surprisingly, no calmodulin (CaM)-like protein able to act as a myosin light chain for MyoJ was identified in the mass spectrometry analysis, except CEN2. This protein shares 30% similarity with MLC1, the myosin light chain of the glideosome complexes (Herm-Götz et al., 2002; Frénal et al., 2014) and disappears from the basal complex upon deletion of MyoJ while MyoJ remains at the posterior cup in absence of CEN2 (Frénal et al., 2017; Lentini et al., 2019). These data suggest that CEN2 could act as a myosin light chain to regulate the function of MyoJ by binding to calcium (Bombardi et al., 2020). The kinase CDPK6 and the S/T phosphatase BCC5 co-localize with MyoJ at the posterior cup, but their depletion did not cause any detectable effect on the constriction of the basal complex or the localization of MyoJ. Yet, it is interesting to note that CDPK6-KO, generated in the type I RH strain, has a mild fitness defect in a competition assay *in vitro*, and, when generated in type III Prugnaud strain, forms fewer tissue cysts in the brain of mice (Long et al., 2016); two phenotypes shared with MyoJ-KO. In addition, deletion of the homologue of CDPK6 in *Plasmodium berghei* causes an unusual circular arrangement of the sporozoites in the oocysts (Tewari et al., 2010). Therefore, it would be interesting to generate a knock-out of BCC5 and CDPK6 to assess the phosphorylation status of MyoJ, BCC1, and CEN2, since we cannot rule out that the auxin-inducible system failed to completely degrade these enzymes. Phosphorylation sites have been identified by mass spectrometry on MyoJ, BCC1, and CEN2 so it is possible that the constriction event is regulated by phosphorylation (Treeck et al., 2011). Finally, in search of the defect that can explain the mild growth defect *in vitro*, we thought to investigate the gliding motility of the BCC1- and MyoJ-KD parasites. We hypothesized that the change of cell shape and cytoskeletal tension due to the non-constricted basal pole could impair the capacity of the parasite to propel itself forward by preventing efficient shear force. Indeed, BCC1- and MyoJ-depleted parasites are less motile than wild-type parasites and when they glide, they perform less helical movements and on shorter distances. These observations could also explain the few tissue cyst counted in the mice infected with MyoJ-KO compared to infection with wild-type parasites (Frénal et al., 2017).

The second phenotype, identified upon BCC5, BCC8, BCC10, and BCC11 depletion, is a loss of rosette organization and synchronicity of intravacuolar division without defect of the basal pole constriction. This was reminiscent of MyoI deletion (Frénal et al., 2017), leading to non-connected parasites, so we analyzed the localization of this myosin in the different cell lines. We noticed a substantial impact on the localization of MyoI with either an accumulation at the basal pole of the BCC5-depleted parasites, a decreased staining in BCC8-KD tachyzoites, or patches of MyoI resembling residual bodies in the case of BCC10 and BCC11 depletion. Therefore, we tested the diffusion of soluble GFP between parasites within the same vacuole using FRAP experiments to determine if the connection was still present. These experiments indicated that depletion of BCC5, located at the posterior cup, and BCC8, BCC10, and BCC11, found below, prevented the exchange of soluble material between the parasites. Although this cytoplasmic bridge is not essential for parasite growth *in vitro*, it would be interesting to check if these proteins’ loss could impact cyst formation *in vivo*. Indeed, these connections are not maintained during differentiation from the fast-dividing tachyzoite to the cyst-forming bradyzoites.

As recently reviewed, the basal complex of the *T. gondii* tachyzoite is critical for several steps of its lytic cycle, including cell division, exchange of soluble factors between intracellular parasites, and possibly uptake of metabolites in extracellular parasites (Gubbels et al., 2022). Our spatial proteomics coupled with labeling reveals that its organization is more complex than previously described, with at least two additional sub-compartments. We hypothesize that the uppermost compartment links the basal complex to the IMC (Vigetti et al., 2022) while the lowest compartment, located at the border between the parasites and the connections, would be dedicated to the formation and/or maintenance of these cytoplasmic bridges allowing cell-cell communication. It remains to determine if BCC8, BCC10, and BCC11 interact to form a specific structure within these nanotubes. Although we identified new proteins of the basal complex, probably, we did not identify all proteins, including some structurally important ones. Further, we did not identify any proteins involved in the segregation of the daughter cells. Still, there are significantly enriched candidates in our mass spectrometry analysis that might be worth investigating. In the future, it would be important to understand how the BCCs are recruited temporally and structurally to build the basal complex and how they are targeted to different sub-compartments. This might be through post-translational modification and especially through palmitoylation because several BCCs have predicted palmitoylation sites in their sequence (**Table 1**), and the protein S-acyl transferase DHHC14 has been identified in our mass spectrometry data.

## Data availability statement

The data presented in the study are deposited in the ProteomeXchange Consortium repository, with the dataset identifier PXD035106.

## Author contributions

KF conceived and designed the project; AAH and KF performed the BioID experiments; J-WD performed the mass spectrometry experiments, and J-WD, CR, and KF analyzed the data; CR, DF, AAH, and KF generated the mutant strains; CR, AAH, DF, and KF analyzed the genotypes and phenotypes; CR performed the confocal imaging and FRAP experiments and analyzed all the collected data; CB and BS performed the EM; IT and PR performed the live imaging of egressing parasites; KF and DRR acquired funding. KF and CR wrote the manuscript and composed the figures; KF, CR, DR, and IT reviewed the manuscript. All authors contributed to the article and approved the submitted version.

## Funding

This study was supported by a Preciput 2019 from the department Sciences Biologiques et Médicales of the University of Bordeaux to KF, a CNRS dotation, and LabEX ParaFrap (ANR-11-LABX-0024) to KF and DR, and by a Ph.D. fellowship from the Ministry of Higher Education, Research and Innovation to CR.

## Acknowledgments

We are grateful to the Bordeaux Imaging Center (BIC) for the use of instrumentation and Magali Mondin and Sébastien Marais for their technical assistance. The BIC is a service unit of the CNRS-INSERM, and Bordeaux University is a member of the national infrastructure France BioImaging supported by the French National Research Agency (ANR-10-INBS-04). We also thank Drs Jean-François Dubremetz, Maryse Lebrun, Sebastian Lourido, Markus Meissner, David L. Sibley, and Dominique Soldati-Favre for providing reagents and/or strains. We are grateful to Dr Sunil Kumar Dogga for his help with the OrthoFinder analysis and to Drs Gaëlle Lentini and Mélanie Bonhivers for critically reading the manuscript.

## Conflict of interest

The authors declare that the research was conducted in the absence of any commercial or financial relationships that could be construed as a potential conflict of interest.

## Publisher’s note

All claims expressed in this article are solely those of the authors and do not necessarily represent those of their affiliated organizations, or those of the publisher, the editors and the reviewers. Any product that may be evaluated in this article, or claim that may be made by its manufacturer, is not guaranteed or endorsed by the publisher.
