# Supplementary material for: Identification of new components of the basal pole of Toxoplasma gondii provides novel insights into its molecular organization and functions
Source: Front Cell Infect Microbiol. 2022 Oct 13;12:1010038. doi: 10.3389/fcimb.2022.1010038 (PMC9613666; doi:10.3389/fcimb.2022.1010038)
Supplement: Supplementary file 1 [file DataSheet_1.pdf]

Supplementary Figures and legends

Figure S1

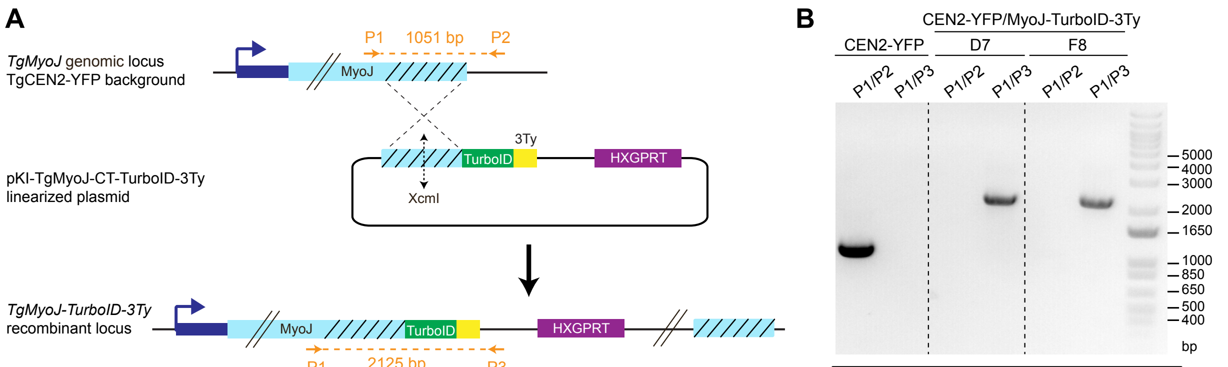

Primer name	5'-3' sequence
P1-4359	CATGGCGGTTCCGACCACAGAATGG
P2-4360	CACTAGGCGCTGGAAGTGTAGCTC
P3-P30A	GTGACACCTGCAAGCCACAGCGG

Figure S1. Strategy used to fuse the TurboID-3Ty to the C-terminus of MyoJ
(A) The integration of the TurboID-3Ty was done by knock-in at the 3' end of the MyoJ coding sequence in the CEN2-YFP cell line. **(B)** Two clones, positive by immunofluorescence (D7 and F8), were analyzed by PCR on extracted gDNA to confirm the site of integration (P1-P3, expected size of 2125 bp) and the clonality (P1-P2, expected size of 1051 bp) compared to the parental CEN2-YFP strain.

Figure S2

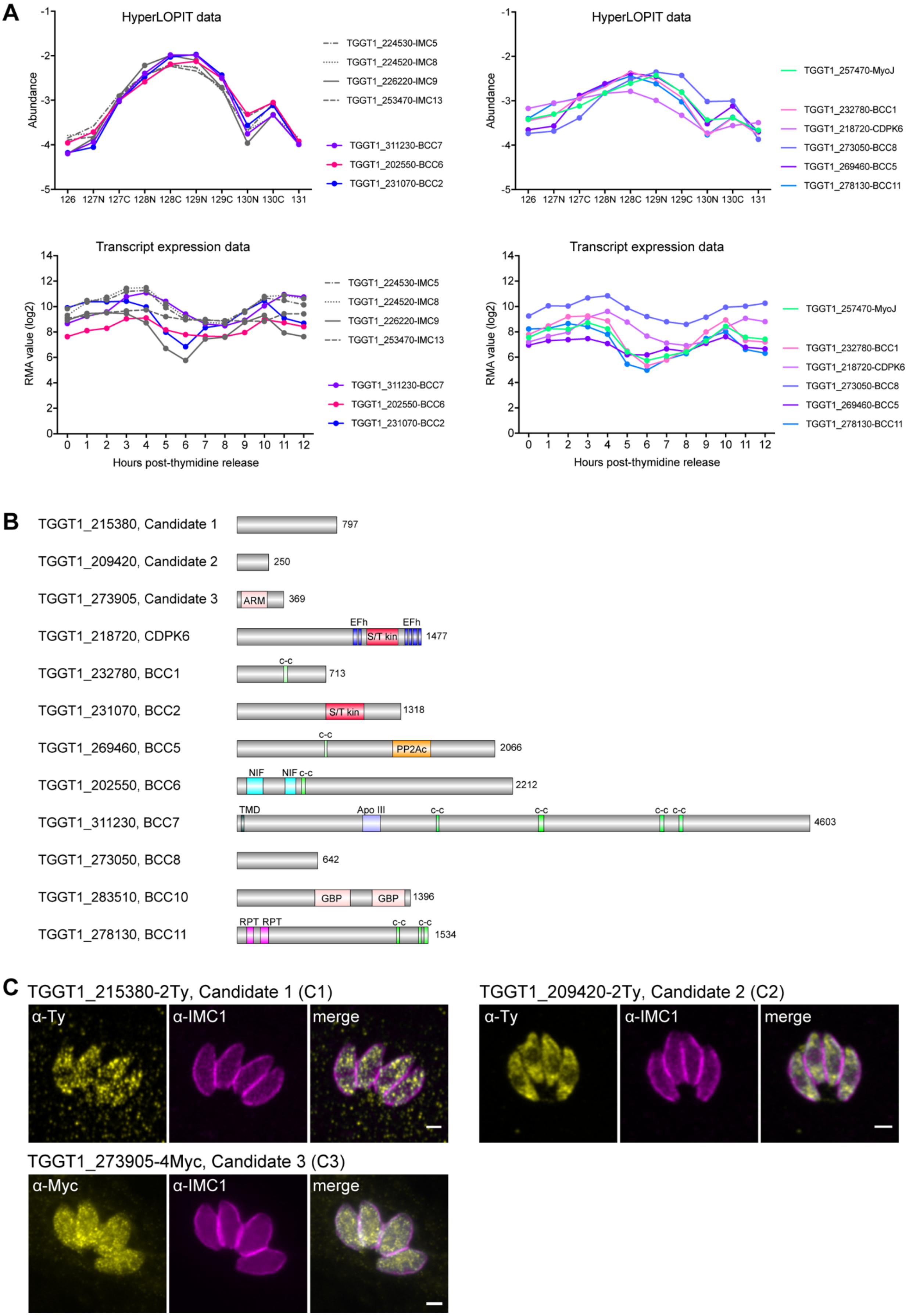
A
HyperLOPIT data
Abundance
126 127N 127C 128N 128C 129N 129C 130N 130C 131
TGGT1_224530-IMC5
TGGT1_224520-IMC8
TGGT1_226220-IMC9
TGGT1_253470-IMC13
TGGT1_311230-BCC7
TGGT1_202550-BCC6
TGGT1_231070-BCC2
TGGT1_257470-MyoJ
TGGT1_232780-BCC1
TGGT1_218720-CDPK6
TGGT1_273050-BCC8
TGGT1_269460-BCC5
TGGT1_278130-BCC11
Transcript expression data
RMA value (log2)
Hours post-thymidine release
B
TGGT1_215380, Candidate 1
797
TGGT1_209420, Candidate 2
250
TGGT1_273905, Candidate 3
ARM
369
TGGT1_218720, CDPK6
EFh
S/T kin
EFh
1477
TGGT1_232780, BCC1
c-c
713
TGGT1_231070, BCC2
S/T kin
1318
TGGT1_269460, BCC5
c-c
PP2Ac
2066
TGGT1_202550, BCC6
NIF
NIF c-c
2212
TGGT1_311230, BCC7
TMD
Apo III
c-c
c-c
c-c c-c
4603
TGGT1_273050, BCC8
642
TGGT1_283510, BCC10
GBP
GBP
1396
TGGT1_278130, BCC11
RPT RPT
c-c
c-c
1534
C
TGGT1_215380-2Ty, Candidate 1 (C1)
α-Ty
α-IMC1
merge
TGGT1_209420-2Ty, Candidate 2 (C2)
α-Ty
α-IMC1
merge
TGGT1_273905-4Myc, Candidate 3 (C3)
α-Myc
α-IMC1
merge

Figure S2. 12 candidates selected from mass spectrometry data analysis

(A) Upper panels: abundance-distribution profiles of basal complex proteins measured in the LOPIT2 experiment (Barylyuk *et al.*, 2020). Lower panels: cell cycle expression profiles (transcripts) of basal complex proteins measured at hour intervals through 12 h post-thymidine release (Behnke *et al.*, 2010). **(B)** Domains and/or motifs predicted in the 12 selected candidates according to SMART (https://smart.embl.de). ApoIII: Apolipophorin-III domain, ARM: armadillo, c-c: coiled-coil, EFh: EF-hand, GBP: guanylate-binding protein domain, NIF: NLI-interacting factor family phosphatase, PP2Ac: Protein Phosphatase 2A catalytic domain, RPT: internal repeats, S/T kin: Serine/Threonine kinase domain, TMD: trans-membrane domain. CDPK: calcium-dependent protein kinase, BCC: basal complex component. **(C)** Three candidates were not localized to the basal pole but rather found in the cytosol (TGGT1_215380 and TGGT1_209420), or cytoplasm (TGGT1_273905) after endogenous tagging. Yellow: protein of interest, magenta: IMC1. Scale bars: 2 μm.

Figure S3

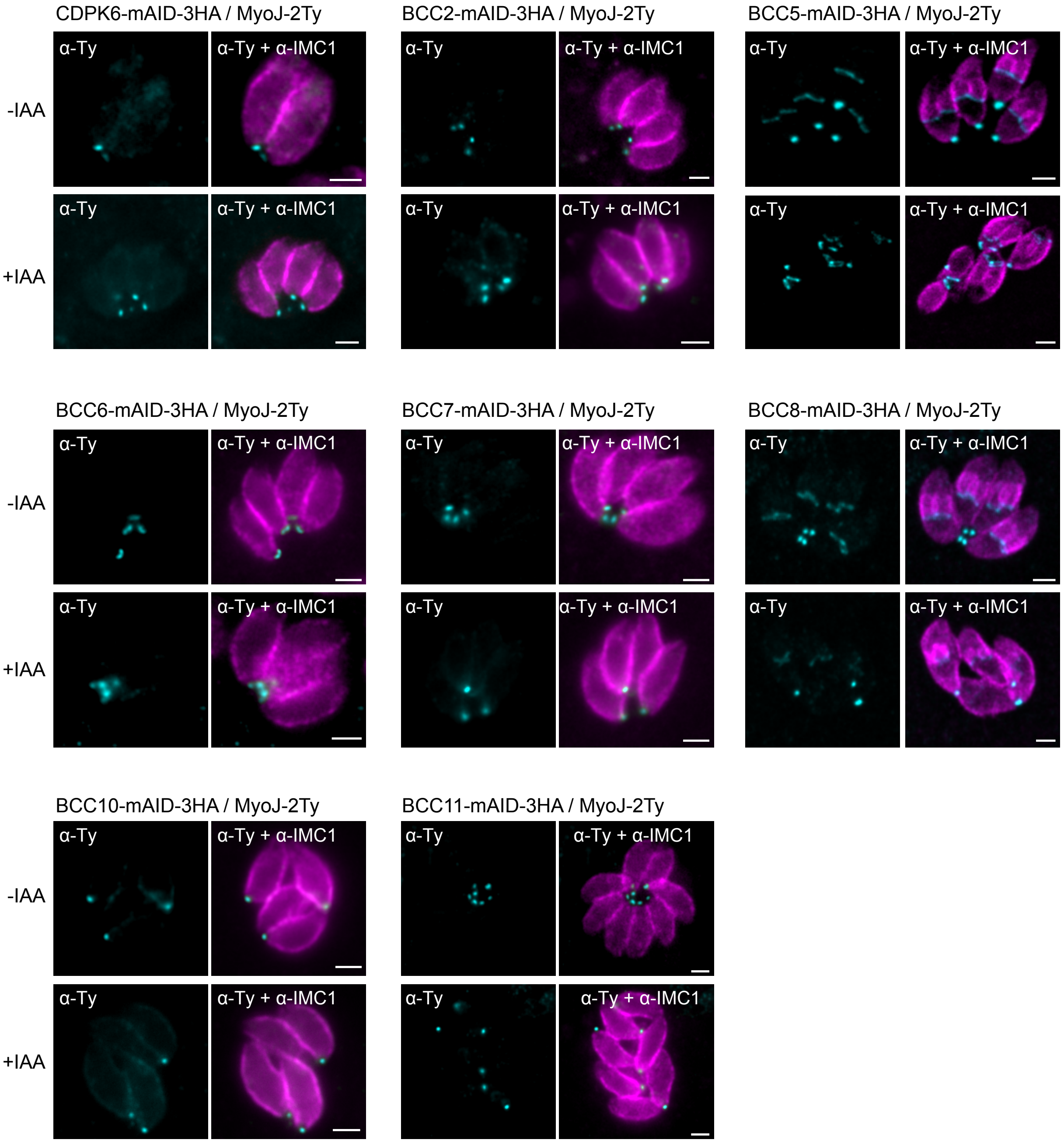

Figure S3. Depletion of CDPK6, BCC2, BCC5, BCC6, BCC7, BCC8, BCC10 and BCC11 does not impair the basal pole constriction

Immuno-staining of endogenously tagged MyoJ-2Ty in the auxin-inducible knockdown of CDPK6, BCC2, BCC5, BCC6, BCC7, BCC8, BCC10, and BCC11 shows that after a 24 h treatment with or without IAA, the basal complex of the parasites remains constricted. Cyan: MyoJ, Magenta: IMC1. Scale: 2μm.

Figure S4

A

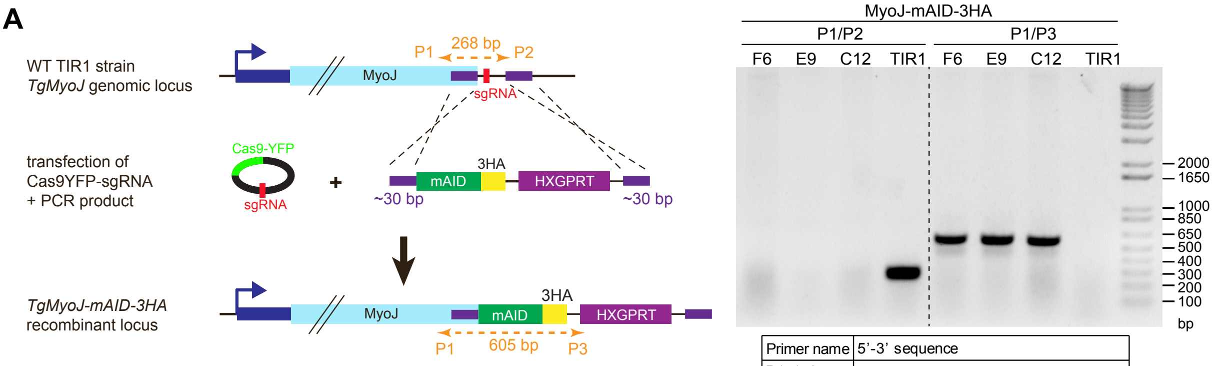

Primer name	5'-3' sequence
P1-1737	CGTGTCTTGTGCTTTCGCAGAG
P2-1738	CACCGGAGTTAGAGCCAGACATG
P3-7139	ATACATTCGCAGACGGATCGT

B

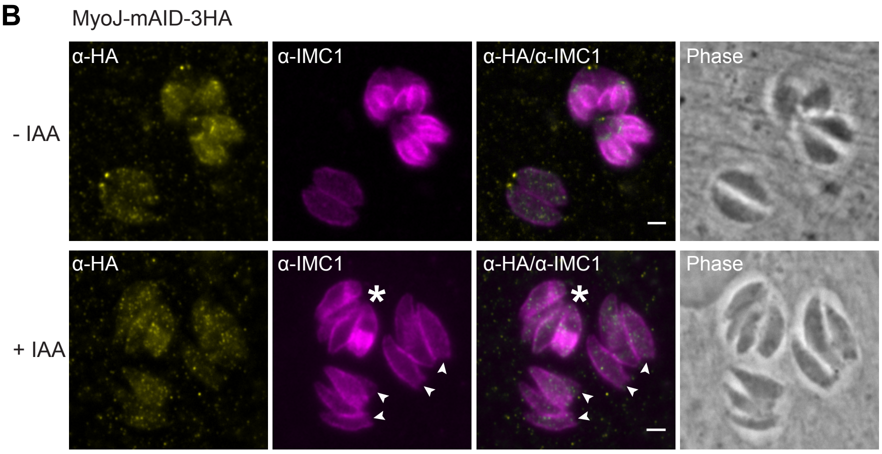

Figure S4. Generation of the MyoJ-mAID-3HA cell line

(A) Integration of the mAID-3HA was done using a specific single gRNA for the Cas9-YFP to target the 3' end of TgMyoJ and enhance the integration of the PCR product carrying ~30 bp of homology with the 3' end of TgMyoJ. Primers used for the sgRNA and the PCR product are listed in Supplementary Table 1. Diagnostic PCRs were performed on gDNA extracted from parental strain (TIR1) and 3 clones of the mutant (C12, E9 and F6), positive by immunofluorescence for HA staining. The correct integration in the targeted locus was checked using primers P1/P3 (expected size of 605 bp) while the clonality was assessed using the primers P1/P2 (expected size of 268 bp). **(B)** Immunofluorescence performed on MyoJ-mAID-3HA cell line after 24 h of growth with or without auxin (IAA). The asterisk shows a vacuole in which the parasites divides asynchronously while the arrowheads point to the larger basal pole of the treated parasites.

Figure S5

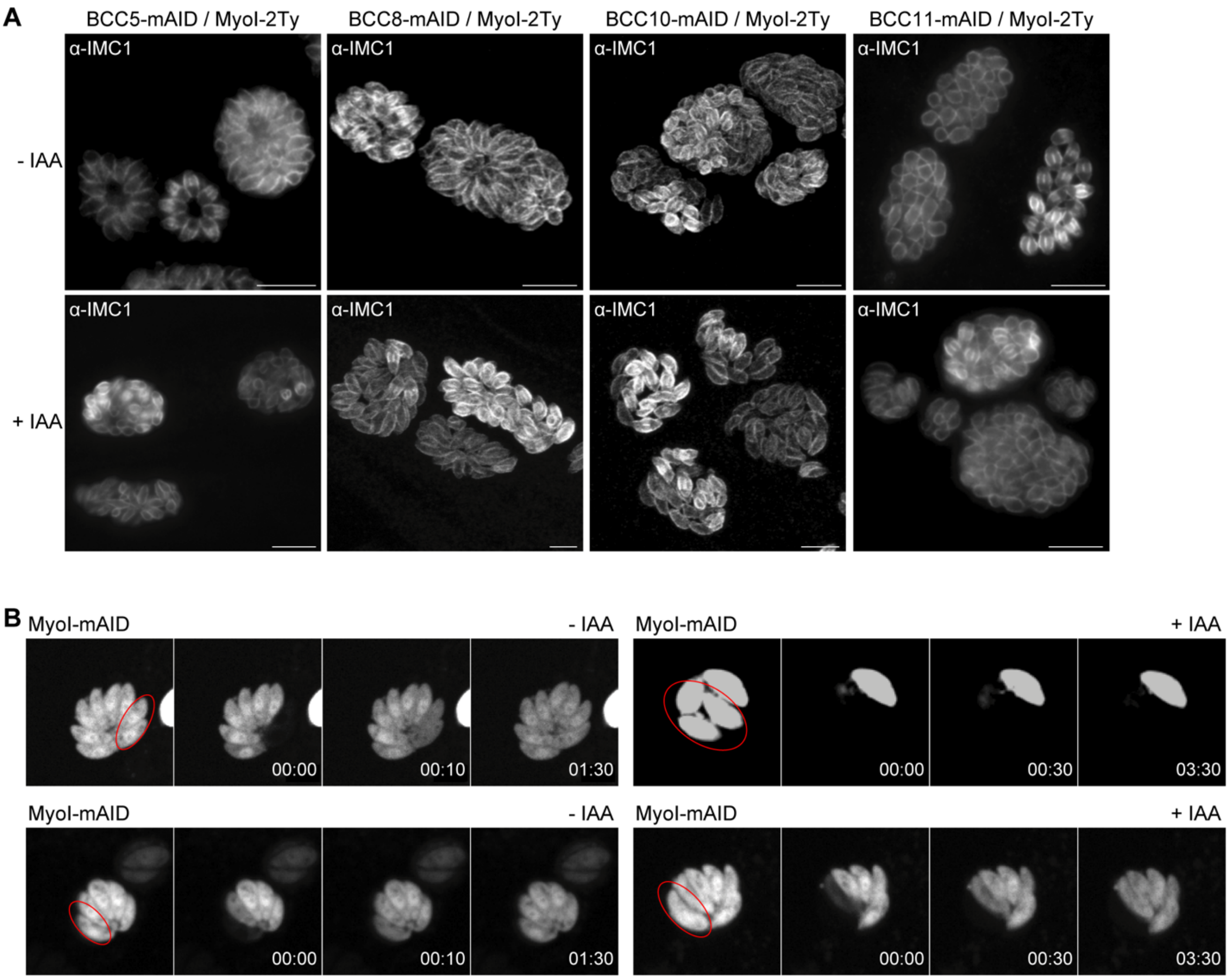

C

	MyoJ-KO	MyoI-2Ty	MyoI-mAID		BCC5-mAID		BCC8-mAID		BCC10-mAID		BCC11-mAID		BCC1-mAID		BCC7-mAID	
IAA (h)	0	0	0	24	0	24	0	24	0	24	0	24	0	24	0	24
n =	7	3	19	22	5	10	8	10	8	10	7	9	14	25	8	4

Figure S5. BCC5, BCC8, BCC10 and BCC11 depleted parasites are impaired in their rosette organization and intra-vacuolar cell-cell connection

(A) The rosette organization and synchronicity of division was assessed by immunofluorescence after a 40-h treatment with or without of auxin (IAA) using the marker IMC1 allowing the visualization of the IMC of mature parasites and growing daughter cells. Scale bars: 10 µm. **(B)** Representative FRAP experiments exemplifying the “connected” (- IAA) and “non-connected” (+ IAA) parasites. **(C)** The table indicates the number of photobleached vacuoles for each strain.

Supplementary Tables

Table S1. List of primers used in this study for cloning and transfections. The restriction sites are underlined, the sgRNA sequences are in red, the homology regions are in blue.

Purpose	Primer ID	5'-3'-Sequence
MyoJ-TurboID	Turbo-8271	GCATGCATCAAAAGACAATACTGTGCCTCTG
	Turbo-8270	CGCCTGCAGGCTTTTCGGCAGACCGCAGACTG
MyoJ-sgRNA	MyoJ-1734	GGCATTTGGTGTCTCCTCTGGTTTTAGAGCTAGAAATAGC
	gRNA-4883	AACTTGACATCCCCATTTAC
MyoJ-mAID / -2Ty PCR	MyoJ-1735	GGCATCCCGAAGCATGGTCGCAGCTGGAAATGGCTAGCAAGGGCTCGGG
	MyoJ-1736	CGTTACGATTTTGTGCATTTCCAACTGCTGTCATACGACTCACTATAGGG
C1-sgRNA	MIP1-1898	AAGTTAGGACGATGGGCTTATTCATG
	MIP1-1899	AAAACATGAATAAGCCCATCGTCCTA
C1-mAID / -2Ty PCR	MIP1-1913	GAAGAGAATGAAGATTTACTGGCAGCAGAAGAGGCTAGCAAGGGCTCGGG
	MIP1-1914	GCCACAGTCAAACTGTCTTTCTACAGAGTTTCCATACGACTCACTATAGGG
C2-sgRNA	MIP2-1900	AAGTTAAAGATGCAAGCGTCGTGTGG
	MIP2-1901	AAAACCACACGACGCTTGCATCTTTA
C2-mAID / -2Ty PCR	MIP2-1915	CAAAACAGGAAGAGGCTCCCCTCAAGATCTGCGGCTAGCAAGGGCTCGGG
	MIP2-1916	CTCCGTTCACTCCGCGCAGGTTCGGCGACTCTCATACGACTCACTATAGGG
KI-MIP3	MIP3-1760	TATAGGGCGAATTGGGTACCGGGCCCGAAAGAGGGACAGAGCGAAAG
	MIP3-1761	GCTAATTAGCTTCTGCTCCAATGCATGCATCGCCAGCATCTTGGCTTGC
BCC1-sgRNA	MIP4-1846	AAGTTAACGCTGCAAGGGTGCAGTTG
	MIP4-1847	AAAACAACTGCACCCTTGCAGCGTTA
BCC1-mAID / -2Ty PCR	MIP4-1848	ACAGGTCGTCGTGATAGAGGTGTCTCCGACAAGGCTAGCAAGGGCTCGGG
	MIP4-1849	CACTCTTATGTGTGCTTTTTGGACGTTGCTCTGATACGACTCACTATAGGG
CDPK6-sgRNA	MIP5-1902	AAGTTGCTCTCGTCGCAACTTCCTTG
	MIP5-1903	AAAACAAGGAAGTTGCGACGAGAGCA
CDPK6-mAID / -2Ty PCR	MIP5-1902	TGGGACGAGTTCGTCGCGTATATGCGCCACGACGCTAGCAAGGGCTCGGG
	MIP5-1903	GGGCTGGCTTGGGCAGAGCGACCCGCAGTGCCATACGACTCACTATAGGG
BCC8-sgRNA	MIP6-1868	AAGTTGCCGTCAACCAGCAGAGCCTGG
	MIP6-1869	AAAACCAGGCTCTGCTGGTTGACGGCA
BCC8-mAID / -2Ty PCR	MIP6-1870	CCTGTCAATCGCACATCTGCTGTGCCAGGTTTCGCTAGCAAGGGCTCGGG
	MIP6-1871	ATTACATCGACTTATTAAATCGACTCTCTGCATATACGACTCACTATAGGG
BCC5-sgRNA	MIP7-1850	AAGTTGCAGGTTAGTCGATGTCGCGG
	MIP7-1851	AAAACCGCGACATCGACTAACCTGCA
BCC5-mAID / -2Ty PCR	MIP7-1852	AAGAACGCGAGAAGAAGGAAGTCCTTCGCAGGTGCTAGCAAGGGCTCGGG
	MIP7-1853	TGCTCACCCCGCCCACTCCTCGTCTCTTCTTCTATACGACTCACTATAGGG
BCC11-sgRNA	MIP8-1904	AAGTTGCTTGTCACCGCCGCAAGTGAG
	MIP8-1905	AAAACTCACTTGCGGCGGTGACAAGCA
BCC11-mAID / -2Ty PCR	MIP8-1920	GCGGACGCAGGAGCCGCGGAAGTGATCGACAAAGCTAGCAAGGGCTCGGG
	MIP8-1921	CATTACGGACGATGATTACAATCAATTCATTGATACGACTCACTATAGGG
BCC7-sgRNA	MIP9-1873	AAGTTGTGAAATGAAGGACGTAATAGG
	MIP9-1874	AAAACCTATTACGTCCTTCATTTCACA
BCC7-mAID / -2Ty PCR	MIP9-1875-	TCGTGGTTCCCGCCGGAAGAAGAGGGCTGCGAGGCTAGCAAGGGCTCGGG
	MIP9-1876	CCACACAGTCTTTCCTAAGCTGATTCTTTCTCAATACGACTCACTATAGGG
BCC6-sgRNA	MIP10-1906	AAGTTGGCCCCAAGCGACGAGAACAG
	MIP10-1907	AAAACTGTTCTCGTCGCTTGGGGCCA
BCC6-mAID / -2Ty PCR	MIP10-1922	GGAGGAAGTAAAGGCAAGGACGCACAAAGGCGCGCTAGCAAGGGCTCGGG
	MIP10-1923	CTCCTTTCCCGCTCTTCTTCGTTCTGTTCTCCATACGACTCACTATAGGG
BCC2-sgRNA	MIP11-1908	AAGTTGGAGATTGCAAGAGCTTCGAG
	MIP11-1909	AAAACTCGAAGCTCTTGCAATCTCCA
BCC2-2Ty PCR	MIP11-1924	GTTGTGTCAGCGGCATCAGCTGACTTCGTATGCGCTAGCAAGGGCTCGGG
	MIP11-1925	ACTGAAGCCACCACGAGGAAGGAATTGCATGATACGACTCACTATAGGG
BCC10-sgRNA	MIP12-1928	AAGTTGTCGTTTCAGGCCGCCCTGCAG
	MIP12-1929	AAAACTGCAGGGCGGCCTGAAACGACA
BCC10-mAID / -2Ty PCR	MIP12-1930	AAATGCTTCCGACAACGGAAATGCACAATCATGGCTAGCAAGGGCTCGGG
	MIP12-1931	CGGTTGCTGGGACGCCACAGGACACCCCTGGAATACGACTCACTATAGGG

Table S2. List of antibodies used in this study

Primary antibodies			
	IFA	U-ExM	WB
anti-streptavidin Alexa 594 conjugated (S32356, Mol. Probes)	1 : 3000		
anti-streptavidin-HRP (RPN1231, Cytiva)			1 : 2 000
anti-Ty IgG1 mouse (clone BB2), (Bastin *et al.* 1996)	1 : 1000		1 : 2000
anti-HA rat (clone 3F10, 11867423001, Roche)	1 : 1500		
anti-HA mouse IgG1 (clone 16B2, 901513, Biolgend)	1 : 1000		1 : 1000
anti-HA mouse IgG2b (clone 12CA5, gift from J-F. Dubremetz)	1 : 1000	1 : 200	
anti-HA rabbit (GTX115044, GeneTex)		1 : 500	
anti-actin mouse, supernatant of hybridoma (Gift from D. Soldati-Favre) (Herm-Götz *et al.* 2002)			1 : 20
anti-GFP rabbit (Living Colors full-length A.v. polyclonal antibody, 632460, Clontech)	1 : 1000	1 : 500	
anti-IMC1 rabbit (Gift from D. Soldati-Favre) (Frénal *et al.* 2014)	1 : 2000	1 : 1000	
anti-GAP45 rabbit (Gift from D. Soldati-Favre) (Plattner *et al.* 2008)	1 : 10000		
anti-acetylated tubulin mouse IgG2b (clone 6-11B-1, T7451, Sigma-Aldrich)		1 : 500	
anti-SAG1 mouse (clone DG52, supernatant of hybridoma) (Bülow et Boothroyd 1991)	1 : 50		
**Secondary antibodies**			
Goat anti-rabbit IgG StarBright™ Blue 700 (12004161, Bio-Rad)			1 : 2500
Peroxidase AffiniPure Sheep anti-mouse IgG (H+L) (515-035-062, Jackson)			1 : 10000
Goat anti-Mouse IgG (H+L) Cross-Adsorbed Secondary Antibody, Alexa Fluor 488 (A11001, Mol. Probes)	1 : 3000		
Goat anti-Mouse IgG (H+L) Cross-Adsorbed Secondary Antibody, Alexa Fluor 594 (A11005, Mol. Probes)	1 : 1000		
Donkey anti-Rabbit IgG (H+L) Highly Cross-Adsorbed Secondary Antibody, Alexa Fluor 647 (A31573, Mol. Probes)	1 : 1000		
Donkey anti-Rat IgG (H+L) Highly Cross-Adsorbed Secondary Antibody, Alexa Fluor 488 (A21208, Mol. Probes)	1 : 1500		
Goat anti-Rabbit IgG (H+L) Cross-Adsorbed Secondary Antibody, Alexa Fluor 594 (A11012, Mol. Probes)	1 : 3000	1 : 500	
Goat anti-Mouse IgG1 Cross-Adsorbed Secondary Antibody, Alexa Fluor 488 (A21121, Mol. Probes)	1 : 1000	1 : 500	
Goat anti-Mouse IgG2b Cross-Adsorbed Secondary Antibody, Alexa Fluor 647 (A21242, Mol. Probes)	1 : 1000	1 : 500	
Goat anti-Rabbit IgG (H+L) Cross-Adsorbed Secondary Antibody, Alexa Fluor 405 (A31556, Mol. Probes)		1 : 500	
Goat anti-Mouse IgG2b Cross-Adsorbed Secondary Antibody, Alexa Fluor 488 (A21141, Mol. Probes)	1 : 2000		

Table S3. Proteomic analysis of BioID experiments performed in duplicate with *Toxoplasma gondii* MyoJ protein tagged with TurboID in tachyzoites

Sheet 1. All the proteins identified by mass spectrometry in the two experiments, Sheet 2. Proteins found in common in the two experiments, Sheet 3. List of the proteins found in common and significantly enriched.

Table S4. Identification of putative orthologues in Apicomplexa

Sheet 1. Accession numbers of the putative orthologues of the 12 selected candidates found using Orthofinder, Sheet 2. Conservation of the 12 selected candidates across the Apicomplexans.

Supplementary materials and methods

Identification of putative orthologues in Apicomplexa

The search for orthologues of our 12 candidates across the apicomplexan phylum was performed with OrthoFinder v2.5.2 (https://github.com/davidemms/OrthoFinder) using default parameters (Emms and Kelly, 2019). The eukaryotic proteomes were downloaded from ToxoDB, PlasmoDB, CryptoDB, PiroplasmaDB and CryptoDB through VEuPathDB (Amos *et al.*, 2022). The putative orthologues identification numbers can be found in supplementary Table 2.

Supplementary files

Movie S1. Natural egress of BCC1-mAID-3HA/MyoJ-2Ty parasites.
Egressing parasites have been recorded during egress and motility by taking 1 image/s over 20 min.

Movie S2. Natural egress of BCC1-mAID-3HA/MyoJ-2Ty parasites after treatment with auxin (IAA) for 48 h.
Egressing parasites have been recorded during egress and motility by taking 1 image/s over 20 min.

Supplementary references

Amos, B., Aurrecoechea, C., Barba, M., Barreto, A., Basenko, E.Y., Bażant, W., et al. (2022) VEuPathDB: the eukaryotic pathogen, vector and host bioinformatics resource center. *Nucleic Acids Research* 50: D898–D911

Barylyuk, K., Koreny, L., Ke, H., Butterworth, S., Crook, O.M., Lassadi, I., et al. (2020) A comprehensive subcellular atlas of the *Toxoplasma* proteome via hyperLOPIT provides spatial context for protein functions. Cell Host & Microbe 28: 752-766.e9.

Bastin, P., Bagherzadeh, Z., Matthews, K.R., and Gull, K. (1996) A novel epitope tag system to study protein targeting and organelle biogenesis in *Trypanosoma brucei*. *Mol Biochem Parasitol* 77: 235–239.

Behnke, M.S., Wootton, J.C., Lehmann, M.M., Radke, J.B., Lucas, O., Nawas, J., et al. (2010) Coordinated progression through two subtranscriptomes underlies the tachyzoite cycle of *Toxoplasma gondii*. PLoS One 5: e12354.

Bülow, R., and Boothroyd, J.C. (1991) Protection of mice from fatal *Toxoplasma gondii* infection by immunization with p30 antigen in liposomes. J Immunol 147: 3496–3500.

Emms, D.M., and Kelly, S. (2019) OrthoFinder: phylogenetic orthology inference for comparative genomics. *Genome Biol* 20: 238.

Frénal, K., Marq, J.-B., Jacot, D., Polonais, V., and Soldati-Favre, D. (2014) Plasticity between MyoC- and MyoA-glideosomes: an example of functional compensation in *Toxoplasma gondii* invasion. *PLoS Pathog* 10: e1004504.

Herm-Götz, A., Weiss, S., Stratmann, R., Fujita-Becker, S., Ruff, C., Meyhöfer, E., et al. (2002) *Toxoplasma gondii* myosin A and its light chain: a fast, single-headed, plus-end-directed motor. *EMBO J* 21: 2149–2158.

Plattner, F., Yarovinsky, F., Romero, S., Didry, D., Carlier, M.-F., Sher, A., and Soldati-Favre, D. (2008) *Toxoplasma* profilin is essential for host cell invasion and TLR11-dependent induction of an interleukin-12 response. *Cell Host & Microbe* 3: 77–87.
